# An optimized single chain TCR scaffold relying on the assembly with the native CD3-complex prevents residual mispairing with endogenous TCRs in human T-cells

**DOI:** 10.18632/oncotarget.8385

**Published:** 2016-03-26

**Authors:** Diana Knies, Sebastian Klobuch, Shao-An Xue, Matthias Birtel, Hakim Echchannaoui, Oezlem Yildiz, Tana Omokoko, Philippe Guillaume, Pedro Romero, Hans Stauss, Ugur Sahin, Wolfgang Herr, Matthias Theobald, Simone Thomas, Ralf-Holger Voss

**Affiliations:** ^1^ Department of Hematology, Oncology, and Pneumology, University Cancer Center (UCT), University Medical Center (UMC) of Johannes Gutenberg University, Mainz, Germany; ^2^ Institute of Immunity and Transplantation, University College London, Royal Free Hospital, London, United Kingdom; ^3^ TRON-Translational Oncology at the University Medical Center of Johannes Gutenberg University gGmbH, Mainz, Germany; ^4^ Biopharmaceutical New Technologies (BioNTech) Corporation, Mainz, Germany; ^5^ Ludwig Institute for Cancer Research (LICR), Lausanne Branch, Epalinges, Switzerland; ^6^ Translational Tumor Immunology Group, Ludwig Center for Cancer Research of the University of Lausanne, Lausanne, Switzerland; ^7^ Research Center for Immunotherapy (FZI), University Medical Center of Johannes Gutenberg University, Mainz, Germany; ^8^ Municipal Clinic Karlsruhe, Karlsruhe, Germany; ^9^ Department of Internal Medicine III, Hematology and Oncology, University Hospital, Regensburg, Germany; ^10^ TCMetrix, Epalinges, Switzerland; ^11^ Center for Interventional Immunology, University of Regensburg, Regensburg, Germany

**Keywords:** human, tumor immunity, T-cells, T-cell receptors, gene therapy, Immunology and Microbiology Section, Immune response, Immunity

## Abstract

Immunotherapy of cancer envisions the adoptive transfer of T-cells genetically engineered with tumor-specific heterodimeric α/β T-cell receptors (TCRα/β). However, potential mispairing of introduced TCRα/β-chains with endogenous β/α-ones may evoke unpredictable autoimmune reactivities. A novel single chain (sc)TCR format relies on the fusion of the Vα-Linker-Vβ-fragment to the TCR Cβ-domain and coexpression of the TCR Cα-domain capable of recruiting the natural CD3-complex for full and hence, native T-cell signaling. Here, we tested whether such a gp100(280-288)- or p53(264-272) tumor antigen-specific scTCR is still prone to mispairing with TCRα. In a human Jurkat-76 T-cell line lacking endogenous TCRs, surface expression and function of a scTCR could be reconstituted by any cointroduced TCRα-chain indicating mispairing to take place on a molecular basis. In contrast, transduction into human TCRα/β-positive T-cells revealed that mispairing is largely reduced. Competition experiments in Jurkat-76 confirmed the preference of dcTCR to selfpair and to spare scTCR. This also allowed for the generation of dc/scTCR-modified cytomegalovirus/tumor antigen-bispecific T-cells to augment T-cell activation in CMV-infected tumor patients. Residual mispairing was prevented by strenghtening the Vα-Li-Vβ-fragment through the design of a novel disulfide bond between a Vα- and a linker-resident residue close to Vβ. Multimer-stainings, and cytotoxicity-, IFNγ-secretion-, and CFSE-proliferation-assays, the latter towards dendritic cells endogenously processing RNA-electroporated gp100 antigen proved the absence of hybrid scTCR/TCRα-formation without impairing avidity of scTCR/Cα in T-cells. Moreover, a fragile cytomegalovirus pp65(495-503)-specific scTCR modified this way acquired enhanced cytotoxicity. Thus, optimized scTCR/Cα inhibits residual TCR mispairing to accomplish safe adoptive immunotherapy for bulk endogenous TCRα/β-positive T-cells.

## INTRODUCTION

T-cell receptors (TCR) are immunglobulin-like folded membrane proteins expressed on CD4^+^/CD8^+^ T-cells with the ability to recognize complexes of processed peptides associated with MHC-molecules on antigen presenting cells (APC). Current strategies in immunotherapy of cancer aim at equipping patients' T-cells with tumor reactive TCRs by e.g. retroviral gene transfer ex vivo and to reinfuse them systemically. In several clinical trials a large benefit for the patient in reducing or even extinguishing tumor burden has been demonstrated [1].

However, the heterologous overexpression of the TCR in T-cells necessitates optimization of the TCR framework to accomplish biochemical inertness against endogenous TCRs. Indeed, since firstly, the TCR is a heterodimer comprising TCRα- and TCRβ-chain and secondly, the retroviral introduction of an exogenous TCR does not override expression of the endogenous TCR, the formation of mixed TCR chain pairing with unpredictable consequences on self-antigen recognition is a distinct possibility [2]. At worst, neoreactivities which end up with autoimmunity [3, 4] may impose severe adverse reactions in adoptive TCR gene transfer-based clinical trials. Thus, major efforts have been invested into the design of TCRs endowed with preferential expression over endogenous TCRs [5-7], the mutual exclusion to interact with endogenous TCRs [8], or high-affinity antigen recognition [9]. Alternatively, endogenous TCRs have been targeted in T-cells *via* sequence-specific siRNA-technology [10], or genomic editing by zinc finger nucleases [11], or TALENs [12].

One common approach relies on the generation of single chain TCR (scTCR)-fragments by covalently bridging the antigen-recognizing V-domains with a 15-20mer of a Glycine/Serine-rich linker (Li) which in theory, inhibits mispairing due to sterical hindrance [13]. Transport to the cell membrane and provision of T-cell signaling upon antigen encounter is accomplished by fusion to the CD3ζ-chain as pioneered by Z. Eshhar for chimeric antigen receptor (CAR)-engineered T-cells [14]. The chimeric immunoreceptor construct assembles to homodimers and operates outside the TCR/CD3-complex which is believed to make mispairing with endogenous TCRs highly unlikely [15, 16]. A recent innovation by this design is the fusion to the TCR Cβ-domain yielding a Vα-Li-Vβ-Cβ 3-domain scTCR which then is coexpressed with a truncated TCRα-chain merely comprising the Cα-domain [17]. Cytotoxicity of scTCR gp100 tranduced T-cells against human melanoma was as efficient as those transduced with the wild type dcTCR *in vitro*. Moreover, they caused a significant delay of tumor growth in NOD/SCID-mice. Notably, this 4-domain topology highly resembles the native TCR architecture so as to accomplish the entire assembly of the TCR/CD3-complex, subsequently leading to a more physiologic proximal T-cell signaling cascade [18]. Cα is crucial for the recruitment of CD3ζ and CD3δε to the TCR/CD3-complex [19]. Chimerization to mouse Cα/Cβ-domains or murinization of a few residues was proven to be necessary to provide for a tight interaction between Cα and Cβ [5, 20, 21]. However, integration into the native CD3-complex may not only restrict expression due to limiting components of the multimeric CD3-complex [22] but may also evoke mispairing [16]. The interaction of TCRα and TCRβ is largely governed by the extensive Cα/Cβ-interface [23], by the individual TCR subfamily V-domains, and also by the antigen-specific CDR3α/β-loops juxtaposed on top of the Vα/Vβ-interface [24]. TCRαβ heterodimer formation is a prerequisite for fully competent TCR/CD3-complex assembly and T-cell signaling. TCRs that prefer to interact with themselves are referred to as ‘strong’ or ‘dominant’ TCRs [25] being able to outcompete ‘weak’ TCRs for binding to the limiting CD3 complex [22].

Here, we prompted us to verify whether such a novel scTCR still tends to mispair with TCRα in a noncompetitive environment of the human leukemia T-cell line Jurkat-76 (J-76) lacking the expression of an endogenous TCR [26], or in the competitive environment of bulk human T-cells. We established a model to unequivocally assign mispairing to the interaction with a full length TCRα-chain, the latter contributing with its CDR3α-loop to antigen recognition: For this, we generated a mutated scTCR CDR3α incapable of binding the cognate antigen and coexpressed it with wild type TCRα of the same antigen specificity serving as a ‘surrogate’ for any endogenous one, to assess reconstitution of antigen recognition. This issue has experimentally been assessed for 2 independent expression systems based on a retroviral [8, 27] or RNA vector [28, 29] for 2 different high-affinity TCRs, a human A2-restricted TCR of the gp100(280-288)-antigen specificity isolated from TILs [30], and a murine A2-restricted TCR of the p53(264-272)-antigen specificity isolated from A2-transgenic mice after peptide immunization [31]. We also mimicked a competitive situation in J-76 by introducing different TCR chain combinations *via* RNA electroporation. Moreover, we quantified the relative expression levels of a ‘strong’ gp100- or p53-specific scTCR and a ‘weak’ CMV-specific dcTCR in antigen-bispecific J-76 and T-cells, respectively, for e.g. the treatment of immunosuppressed CMV^+^ leukemia patients after bone marrow transplantation [32]. TCR-engineered T-cells were tested for their structural avidities in multimer-binding by flow cytometry, and for functional avidities by IFNγ-secretion, cytotoxicity, or proliferation. We also assessed the magnitude of mispairing for saturating amounts of pulsed peptide *versus* endogenous processing of full length antigen following RNA electroporation in autologous iDCs as target cells. Finally, we aimed at strengthening V-domain pairing by the design of a novel disulfide bond into a scTCR-fragment [33] so as to eradicate traceable mispairing with any TCRα.

## RESULTS

### A human 3-domain scTCR gp100(280-288) mispairs with human TCRα in human Jurkat-76 cells devoid of endogenous TCRs

The endogenous TCRα/β-chain deficient Jurkat-76 (J-76) leukemia T-cell line [26] was used to perform TCR mispairing studies. Absence of surface expression of TCRs was confirmed, also for the TCR-associated CD3-complex (Suppl. Figure 1A). Moreover, RNA electroporation [34] of a single TCRα- or TCRβ-chain did not reconstitute pan TCR-expression which might have taken place by pairing with an endogenous TCRβ- or TCRα-chain proving that the genomic defect affected both chains. In contrast, introduction of both TCR-chains, a wild type TCRαβ gp100, or a TCRαβ pp65, and of notice, ‘mispaired’ TCRα gp100 with TCRβ pp65 (and vice versa) led to pronounced human (Hu) pan TCR- or TCR-subfamily-specific staining (Suppl. Figure 1B/1C).

Hence, J-76 cells allow for the unbiased characterization of molecular interactions between any introduced TCR-chains without interference from endogenous TCRαβ counterparts. Therefore this system is also suited for pairing analysis between an introduced scTCR and TCRα-chains of any antigen specificity. The design and nomenclature for all un/modified TCR constructs used here are outlined in Suppl. Figure 1D. Since the scTCR framework to be scrutinized is of the domain order Vα-Li-Vβ-Cβ [17] mispairing with a TCRβ-chain was, as expected, experimentally ruled out (data not shown).

**Figure 1:**
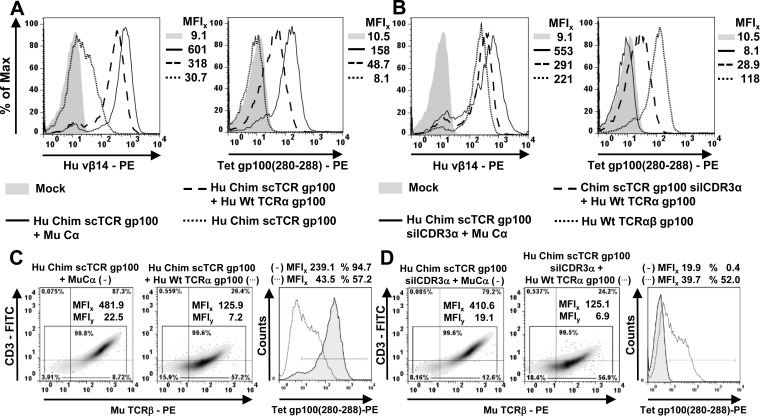
Mispairing of a human scTCR gp100 with a full length human TCRα gp100-chain takes place in Jurkat-76 **A.**/**B.** 5 × 10^6^ J-76 cells were electroporated with 5μg RNA coding for different scTCR gp100 constructs and mouse Cα, the latter required for surface expression. After 12 hours, TCR expression was analyzed cytofluorometrically either with a subfamily-specific antibody Vβ14 or by tetramer (Tet) gp100(280-288) -staining. Mouse Cβ-chimerized (Chim) scTCR gp100 was analyzed for a potential mispairing with Hu Wt TCRα gp100. TCRα operates as a ‘surrogate’ for any (endogenous) TCRα and hence, as a ‘sensor’ of mispairing. The mean fluorescence intensity (MFI) for every specimen is indicated. **C.**/**D.** A corresponding panel of TCR gp100 constructs as in (A) were introduced into J-76 *via* retroviral gene transfer and normalized for gene expression by drug-selection. Hu Chim scTCR gp100, either coexpressed with Mu Ca (left, (−)) or Wt TCRα gp100 (middle, (…)), was analyzed for its surface expression with an anti-mouse TCRβ-specific antibody (density plots) or antigen-specific multimer binding (overlay histogram), functionally unresponsive or so-called ‘silenced’ Hu Chim scTCR gp100 silCDR3α correspondingly in D. Experiment was performed twice.

We used TCR RNA electroporation to introduce high-affinity TCR gp100 [30] coding sequences into J-76 [28, 34] which enables fast expression within hours in a quantitative manner. A prerequisite for scTCR expression according to our design was the coexpression of the mouse (Mu) Cα-domain [17] which triggers the recruitment of the CD3 subunits obligatory for cell surface expression as shown for subfamily- or antigen-specific multimer-staining (Figure 1A, MFIs 601, 158). Additionally, human scTCR gp100 needed to be chimerized (chim) to mouse Cβ so as to exploit stronger murine Cα/Cβ-pairing and interaction with human CD3 [5]. ScTCR gp100(280-288) alone elicited a weak signal in TCRβ-specific Vβ14-staining (MFI 30.7) and no specific signal in antigen-specific multimer-staining (MFI 8.1). The expression of scTCRgp100 + TCRα gp100 led to a profound Vβ14-surface expression (MFI 318) and most importantly, antigen recognition (MFI Tet 48.7) which wasn't expected to take place to such an extent. Hence, the fused 3-domain scTCR gp100 was able to interact with TCRα gp100 of the same antigen-specificity and suggested, that the single chain configuration did not prevent mispairing on a molecular basis.

Next, we mutated the CDR3-region of the scTCR Vα-domain to S109Q according to IMGT nomenclature [35] to eliminate or ‘silence’ (sil) antigen recognition (silCDR3α) without affecting expression (Figure 1B), thereby generating a functionally unresponsive scTCR. Hypothetically, this allowed to unambiguously answer the question whether a (silenced) scTCR is able to pair with the full length TCRα-chain (i.e. Vα + Cα, herein referred to as ‘TCRα-mispairing’) of the same antigen-specificity to restore antigen recognition in trans (i.e. from 2 polypeptides). In general, TCRα-chains of the same antigen specificity turned out to be a ‘sensor’ of mispairing by means of multimer stainings or effector function and therefore, will be referred to as a ‘surrogate’ for any (i.e. human endogenous) TCRα-chain. More indirectly, TCRβ-positivity of a (non-silenced) scTCR in the absence of multimer-binding suggests also an identical mechanism of TCRα-mispairing since both domains of the latter, Vα and Cα, seemingly mispair (e.g. Figure 4C). Alternatively, a (non-silenced) scTCR may just need to pair with the Cα-domain of an unrelated TCRα (referred to as ‘TCR Cα-mispairing’) to preserve antigen-recognition in cis (i.e. from the scTCR polypeptide alone). We supposed, that partial unfolding and/or domain dissociation of a scTCR-fragment (i.e. Vα-Li-Vβ) as observed for scFv-fragments (V_H_-Li-V_L_) [36], may allow this side reaction.

Mutation of CDR3α did not impair expression of the scTCR (MFI 553) while antigen recognition was abolished (MFI 8.1). Moreover, expression of scTCR gp100 silCDR3α + Cα was even higher than for a wild type human double chain TCR (MFI 221) which could be attributed to the murinized C-domains of scTCR gp100 [17]. Again, coexpression of a full length TCRα gp100 led to a marked Vβ14-expression (MFI 291). Most importantly, multimer binding could still be restored in part (MFI 28.9) by TCRα which clearly proved that scTCR gp100 silCDR3α has the potential not only to molecularly associate with the full length TCRα gp100-chain, but also to restore antigen recognition in trans despite the presence of a bulky linker-attached Vα-domain. The higher level of antigen recognition of non-silenced scTCR gp100 + TCRα in comparison to scTCR silCDR3α + TCRα (MFI, 48.7 *vs* 28.9) may likely arise from mispairing caused by both, TCRα- and also but less by TCR Cα-mispairing.

Pairing of scTCR gp100 with the homologous TCRα -chain was also studied in a clinically more relevant retroviral expression system in J-76 (Figure 1C/1D). Transduced Jurkat-76 were drug-selected and expanded to normalize TCR expression [8]. Mu TCRβ-expression of scTCR gp100 + Cα (MFI_x_ 481.9) and scTCR gp100 silCDR3α + Cα (MFI 410.6) was in the same range (Figure 1C *vs* 1D) while for the latter tetramer binding dropped down to background (MFIs 19.9 *vs* 239.1). Moreover, increase in TCR expression correlated with elevated amounts of CD3 surface expression while scTCR gp100 alone failed (data not shown). Again, coexpression of TCRα gp100 proved to partially restore tetramer binding (scTCR + TCRα, MFI 43.5; scTCR silCDR3α + TCRα, MFI 39.7) and in addition, the transport of CD3 to the cell surface (MFI_y_ 7.2, 6.9).

Murinization of human double chain TCRs [5] or single chain TCRs [17] in their TCR C-domains supported TCR expression by favourable chain pairing due to distinct species-specific amino acid interactions at their C-domains' interface [20]. According to our previous findings, mispairing of a chimerized scTCR should be more pronounced with a murinized human TCRα than with a full length human one. To test this, we combined Hu Chim scTCR gp100 with either a human or murinized TCRα gp100 or TCRα pp65-chain by RNA electroporation into J-76 followed by expression analysis and functional validation in a peptide-titrated IFNγ-Elispot assay (Suppl. Figure 2A). Vβ14-staining, and of note, tetramer-positivity and IFNγ-secretion were indeed higher for the murinized TCRα gp100-chain. Additionally, a higher gp100 tetramer-positivity for Chim scTCR gp100 in combination with unrelated Chim TCRα pp65 substantiated the proposed mechanism of TCR Cα-mispairing.

### A mouse 3-domain scTCR p53(264-272) mispairs with human and mouse TCRα in human Jurkat-76

Next, we assessed the amount of hybrid TCR formation for a murine high-affinity scTCR p53(264-272) originating from A2-transgenic mice [31] which followed the same design of a scTCR gp100 + Cα (Suppl. Figure 1D). J-76 cells were retrovirally transduced with scTCR p53 along with Mu Cα or species-related or unrelated TCRα-chains, respectively (Suppl. Figure 2B). Functionally unresponsive (i.e. CDR3α D109A) scTCRp53 elicited exclusively TCRα-mispairing (not TCR Cα-mispairing) in particular with species-related mouse but also with human TCRα-chains. Only the provision of Mu Cα or TCRα p53 led to a recovery in multimer-binding, which was also prominent in an IFNγ -secretion assay (Suppl. Figure 2C). The order of mispairing is in line with experiments conducted with the functionally competent scTCR p53 (corresponding data only shown for T-cells in Figure 4C). Taken together, by taking advantage of a silencing mutation in TCR CDR3α our results clearly demonstrated the incidence of TCRα-mispairing with a 3-domain single chain TCR for a (chimerized) human and a mouse scTCR on a molecular basis in the absence of potentially interfering endogenous TCRα/β-chains.

### Competition of TCR α-chains (i.e. Cα *versus* TCRα) for binding to a scTCR: Cα is able to compete with any TCRα depending on their intrinsic competitive strengths

**Figure 2:**
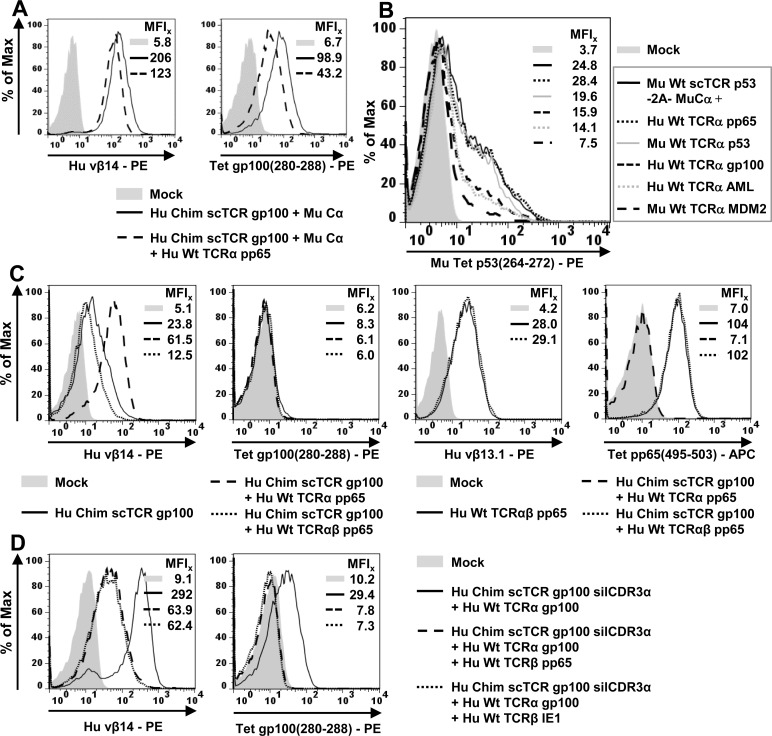
Competitive TCR-chain pairing analyses in Jurkat-76 **(A./B.)** Competition of TCRα and Cα for binding to scTCR gp100. **A.** 5 × 10^6^ J-76 cells were RNA-electroporated with 5μg RNA coding for Cα, scTCR gp100 and TCRα pp65. TCR expression was analyzed by flow cytometry as outlined in Figure 1. **B.** Jurkat cells were retrovirally transduced with scTCR p53-F2A-Cα and TCRα of different antigen-specificities and species-origins, drug-selected, expanded, and analyzed by flow cytometry. (**C**./**D**.) Competition of TCRβ and scTCR for binding to TCRα. The RNA-electroporation experiment was performed as described in (A). **C.** Chimerized scTCR gp100 was coexpressed with TCRαβ of the pp65(495-503)-specificity and tested for Vβ14 (TCR gp100)- or Vβ13.1 (TCR pp65)-, and tetramer gp100- or pp65-positivity. **D.** Functionally unresponsive scTCR gp100 silCDR3α was coexpressed with unmodified TCRα gp100 and different antigen-unrelated CMV (pp65 or IE1)-specific TCRβ-chains. Experiment was performed twice.

In human T-cells, introduced Cα will have to compete with endogenous TCRα for binding to a scTCR. To mimic the competitive situation scTCR gp100 was coelectroporated with Cα and Hu Wt TCRα pp65 in J-76 cells (Figure 2A). The presence of TCRα did not affect the expression of scTCR gp100 significantly (Vβ14, MFI 123) when compared to the expression in the absence of the competitive chain (MFI 206). Therefore, TCRα pp65 seemingly competed only weakly for binding to Chim scTCR gp100. However, it is difficult to discern, whether the observed Vβ14-positivity is due to pairing of the scTCR to Cα or TCRα. Hence, only recovery of antigen binding would give an unbiased answer towards the preferred interaction: Notably, the presence of TCRα did only moderately affect tetramer binding (Tet, MFI 43.2) in comparison to the absence of TCRα (MFI 98.9). Thus, although TCRα is able to pair with the scTCR *via* 2 domains, namely Cα/Cβ and Vα/Vβ at the expense of displacing Vα of the scTCR, pairing with Cα is the more likely reaction presumably because i) both C-domains were murinized characteristic of an exceptionally favorable interdomain affinity [5] and ii) this thermodynamically obviates the need to displace Vα of the scTCR.

We assessed this relationship in more detail for a mouse single chain TCR p53 in a retroviral vector system for which various human TCRα-chains of different antigen specificities were available (Figure 2B). The scTCR p53 was encoded together with Mu Cα on a single retroviral vector *via* a self-processing element F2A [37]. This plasmid was transduced alone or in combination with either a human pp65(495-503)-, gp100(280-288)-, AML [38]-, or murine p53(264-272)-, MDM2(81-88) TCRα-encoding plasmid and normalized in protein expression by combined drug-selection. Coexpression of TCRα pp65 did not affect multimer staining (MFI 28.4) in relation to scTCR p53-F2A-Cα alone (MFI 24.8). As shown before, TCRα pp65 was poorly able to outcompete Cα. TCRα p53(264-272) which behaved as a normalization control here, reduced expression only slightly (19.6) as to be expected. TCRα gp100-chain exhibited a lower tetramer positivity (MFI 15.9) denoting a somewhat more pronounced competition than TCRα pp65. Coexpression of a human AML-reactive TCRα further decreased tetramer binding a little (MFI 14.1). Mouse TCRα MDM2 turned out to be the most potent competitor (MFI 7.5) presumably due to the higher interaction forces between mouse Cα/Cβ as discussed before and hypothetically, also between mouse Vα/Vβ. Importantly, none of the scrutinized TCRα-chains were able to entirely suppress pairing with Cα. This may have important implications for gene therapy in future scTCR + Cα - based clinical trials. The rank order of mispairing behaviour in the presence of Cα and any TCRα could be recovered in an peptide-titrated IFNγ-ELISA (data not shown): Even in the presence of the best suppressor of scTCR p53/Cα - expression, mouse TCRα MDM2, substantial amounts of IFNγ upon p53(264-272) antigen encounter were secreted.

### Competition of TCR β-chains (i.e. TCRβ *versus* scTCR) for binding to TCRα: TCRβ dominates binding to TCRα to assemble to a native TCRαβ

We stated in the previous section that a CMV-specific TCRα pp65-chain is weakly competing for binding to both, a scTCR gp100 and scTCR p53 (Figure 2A/2B) in the presence of Cα. Next, we asked whether such a ‘weak’ TCRα-chain is biased to predominantly interact with its designated binding partner, TCRβ pp65, or in other words, whether TCRβ preferentially binds to TCRα rather than a scTCR. In RNA electroporation experiments with J-76, TCRα pp65 is able to interact with a scTCR gp100 in the absence of Cα (Figure 2C, MFI 61.5). Since TCRα- and not TCR Cα-mispairing apparently took place this combination did not elicit any tetramer-positivity (MFI 6.1). When supplying TCRβ pp65 Vβ14 expression of scTCR gp100 dropped down to background as of scTCR alone (MFI 12.5), the same was true for tetramer staining (MFI 6.0). Obviously, TCRβ pp65 is able to entirely capture TCRα pp65 and to withdraw it from scTCR gp100. Most importantly, TCRβ pp65-specific Vβ13-staining and in particular, CMV(495-503) tetramer-positivity of TCRαβ pp65 in the presence of scTCR gp100 gave rise to a level (MFI 102) comparable to TCRαβ pp65 in the absence of scTCR gp100 (MFI 104). This highlights, that TCRβ pp65 entirely outcompeted scTCR for binding to its designated counterpart, TCRα pp65-chain, an observation that could also be confirmed in an IFNγ-Elispot-assay (data not shown).

However, this might merely reflect a bias towards chain pairing of thymically coevolved dcTCR chains on a clonal level. To address this argument, we took advantage of the S109Q-silenced scTCR gp100 to verify whether Hu TCRα gp100 would either restore gp100 antigen recognition or would also be captured by any unrelated coexpressed TCRβ thereby abolishing gp100 antigen binding. We used TCRβ pp65(495-503) or TCRβ IE-1(316-324) specific for late or early CMV-antigens, respectively (Figure 2D). The coelectroporation of scTCR gp100 silCDR3α and TCRα gp100 into J-76 proved high Vβ14 (MFI 292)- and tetramer-staining (MFI 29.4) as shown before (Figure 1). Cotransfection of scTCR gp100 silCDR3α/TCRα gp100 with TCRβ pp65 or TCRβ IE1-chain lowered Vβ14-staining (MFI 63.9, 62.4) and eliminated any tetramer-positivity (MFI 7.8, 7.3). Therefore, TCRβ-chains in general seem to dominate binding to TCRα gp100 over binding of the latter to scTCR gp100. This implicates that mispairing of a scTCR with TCRα in the presence of any competing TCRβ is rather unlikely to occur.

### Dual specificities of Jurkat-76 coexpressing a single chain TCR gp100 and a double chain TCR pp65

Donor lymphocyte infusion (DLI) takes into consideration the adoptive transfer of dual-specific T-cells carrying a tumor-associated antigen (TAA)-specific engineered TCR along with a CMV-specific endogenous TCR to simultaneously treat refractory leukemias and CMV-reactivation in immunosuppressed patients after bone marrow transplantation [32]. Also, virus/TAA-bispecific T-cells hold great promise to operate more efficiently and to persist longterm in tumor patients due to the long-lasting stimulus by latent viruses such as EBV [39]. In a proof of concept, we initially tested in J-76 the option to coexpress a tumor-reactive scTCR gp100 + Cα and a CMV-specific dcTCR pp65, the latter one which mimics the endogenous TCR pp65 in a CMV-specific T-cell population isolated from a CMV-positive donor. From previous competition experiments we knew that firstly, Cα (and not TCRα) predominantly associates with scTCR (Figure 2A/2B) and secondly, that TCRβ (and not scTCR) dominates association with TCRα (Figure 2C/2D). In theory, these features should allow for the simultaneous expression of a scTCR and a dcTCR in a hematopoietic cell. Depending on the competitive strength of TCRα and TCRβ, either the scTCR or the dcTCR should prevail the relative frequencies of their expressions.

**Figure 3:**
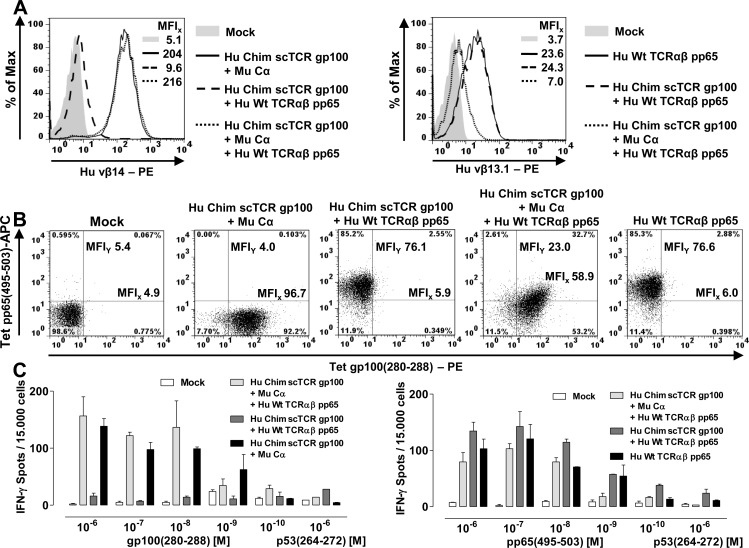
Dual specificities of a scTCR gp100 and a dcTCR pp65 in Jurkat-76 5 × 10^6^ J-76 cells were electroporated with 5μg RNA coding for Cα, or TCRα, TCRβ of the CMV-specificity, or Chim scTCR of the gp100-specificity. **A.** Single or combined expression of Chim scTCR gp100 + Cα and dcTCRαβ pp65 was analyzed cytofluorometrically either with a TCR subfamily-specific antibody vβ14 (gp100, left overlay) or vβ13.1 (pp65, right overlay). **B.** The expression of both TCRs as described in (A) were quantified (MFIs in both dimensions) by pp65(495-503)- or gp100(280-288)-specific tetramer stainings. **C.** 20h-ELIspot assay against dose-dependently peptide loaded T2 for an E:T-ratio = 0.3:1 using gp100- and/or pp65-specific TCR-engineered responder cells as described in (A). Data are shown as mean + SD of duplicates.

Electroporation of scTCR gp100 + Cα gave a robust Vβ14 expression in J-76 (Figure 3A, MFI 204). Transfection of solely scTCR along with dcTCRαβ pp65 reduced expression as expected (MFI 9.6), while the addition of Cα led to a marked expression of scTCR + Cα in the presence of dcTCRαβ pp65 (MFI 216) comparable to scTCR + Cα alone. Vβ13.1-expression of dcTCRαβ pp65 (MFI 23.6) disappeared not before providing both, scTCR gp100 and Cα (MFI 7.0). ScTCR gp100 alone was not able to reduce expression of dcTCR pp65 (MFI 24.3). While when coexpressing both TCRs, scTCR gp100 + Cα was as prominent as the separately expressed one (MFIs 216 *vs* 204), dcTCRαβ pp65 was much less expressed (MFIs 7 *vs* 23.6). This impressively shows that dcTCR pp65 is more susceptible for being outcompeted by scTCR + Cα than vice versa and underlines the weak competitive strength of not only monomeric TCRα pp65 (Figure 2A/Figure 2B) but also of the heterodimeric TCRαβ pp65. This could be illustrated in a 2-dimensional antigen-dependent flow cytometry analysis more vividly (Figure 3B): The tetramer-staining demonstrated strong expression of both, scTCR gp100 + Cα (MFI 96.7) and dcTCRαβ pp65 (MFI 76.6) as long as they were expressed separately. The combined expression of both TCR formats led to a dominant expression of the scTCR gp100 + Cα to more than half of the solitary expressed scTCR (i.e. MFIs 58.9/96.7 = 0.6) while expression of the dcTCRαβ pp65 decreased to less than one third of the separately expressed one (i.e. MFIs 23.0/76.6 = 0.3). This observation could be reasonably well retrieved from an IFNγ-Elispot-assay (Figure 3C). Noteworthy, IFNγ spot formation for both TCR formats and specificities was roughly in the same range and as high as for the related TCRs expressed separately despite their differences in expression.

### Mispairing of a 3-domain scTCR with human TCRα occurs with lower frequencies in human T-cells

**Figure 4:**
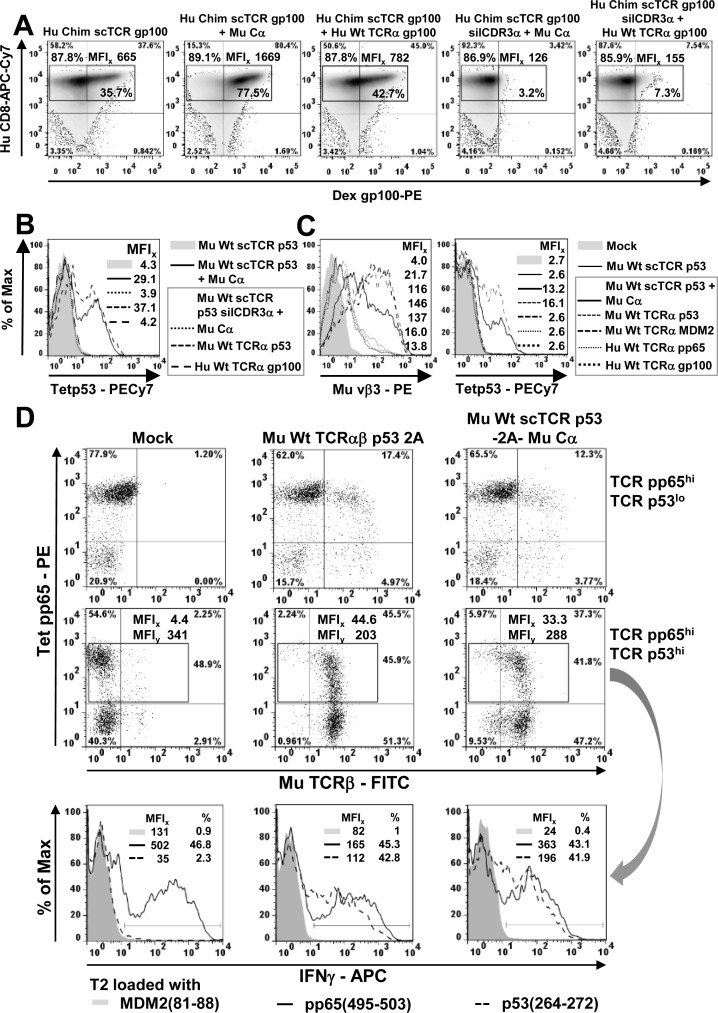
Mispairing of a scTCR with human TCRα takes place to less amounts in human T-cells **A.** 4-10 μg of RNA encoding Cα, human TCRα gp100, or different scTCR gp100-constructs without or with the mutation silCDR3α S109Q to eliminate antigen recognition, were electroporated into MACS-purified human CD8^+^ T-cells and 20 h later analyzed for dextramer gp100(280-288)-positivity in flow cytometry to assess TCRα-mispairing. The overall frequency and MFI of multimer-stained T-cells in gated region is indicated outside the gate in bold, the frequency of multimer-positive T-cells is indicated inside in bold. Multimer-staining was performed twice. **B.** Bulk human T-cells were retrovirally transduced with different scTCR p53 (+/− silCDR3α D109A), Cα, or different TCRα-chains encoded on separate plasmids, normalized in TCR expression *via* drug-selection, expanded for at least a single 2-weekly CD3/CD28-beads stimulation and analyzed in tetramer p53(264-272)-binding to assess TCRα-mispairing. **C.** Bulk human T-cells were retrovirally transduced with wild type scTCR p53, Cα or antigen-un/related TCRα-chains encoded on separate plasmids and analyzed for scTCR p53 expression (vβ3) and antigen-recognition (tetramer p53) to assess TCR Cα-mispairing. Generation of CMV pp65/TAA p53-bispecific T-cells. **D.** Pure pp65-specific oligoclonal T-cell populations were obtained from CMV-positive donors by repetitive peptide stimulation. DcTCRα/β- and scTCR/Cα p53-encoding genes, respectively, were coupled *via* F2A on a single plasmid carrying an IRES-puromycin cassette, and, along with a mock-control (i.e. empty vector), retrovirally introduced into CMV^+^ T-cells (top row), drug-selected to normalize TCR p53 expression and expanded (middle row). A fraction of all T-cells loose TCR CMV-expression after selection with puromycin presumably due to drug-susceptibility. The resulting TCR pp65/p53-double positive (gated) T-cell subsets were assessed for specific cytokine production after coculture with peptide-pulsed T2-cells as indicated by means of intracellular IFNγ-staining (bottom row). This experiment is one out of 2 similar experiments.

Next, we assessed the propensity of scTCR gp100 and p53 to mispair with polyclonal endogenous TCRs in primary human T-cells (Figure 4). We assumed that the natural TCRs would interfere with pairing events on a broader scale than in the TCR-negative J-76 host cell. We RNA-electroporated [28] chimerized scTCR gp100 into bulk human CD8^+^ T-cells (Figure 4A) and observed a modest multimer positivity (35.7 %) of low intensity (MFI 665) presumably originating most-likely from TCR Cα-mispairing as this also became apparent in J-76 (Figure 1A *vs* 1B). In this regard, the mock control Hu Chim scTCR gp100 silCDRα + Cα (3.2 %) confirmed the specificity of the dextramer. Coexpression of the mouse Cα-domain gave rise to a high frequency (77.5 %) and intensity (MFI 1669) in multimer staining which emphasizes that Cα is capable of interacting with scTCR gp100 in the presence of a vast excess of competing polyclonal endogenous TCRα-chains.

Coelectroporation of cognate TCRα gp100 conferred moderate antigen recognition of 42.7 % (MFI 782) again reflecting TCR Cα-mispairing with endogenous and here additionally, exogenous TCRα. As expected, cognate antigen recognition could be eliminated by introducing the S109Q-mutation into Vα CDR3 (3.2 %, MFI 126). Most importantly, the coexpression of TCRα gp100 in place of Cα revealed a minor but clearly discernible fraction of T-cells carrying a mispaired scTCR gp100 silCDR3α + Hu TCRα gp100 (7.3 %, MFI 155). Thus, a low amount of mispairing of scTCR gp100 with TCRα gp100 became imminent in the competitive environment of TCR polyclonal T-cells and hence, will be referred to as ‘residual mispairing’. We hypothesize that beside TCRα gp100 as a ‘surrogate’ for any TCRα-chain mispairing of arbitrary endogenous TCRα-chains of an appropriate interchain affinity is also likely to occur. Worth mentioning, that its low frequency is in line with data obtained in J-76, that murine Cα successfully outcompeted endogenous TCRα for binding to chimerized scTCR (Figure 2A/Figure 2B) and that the majority of TCRα-chains tended to assemble with TCRβ to double chain TCRαβ (Figure 2C/Figure 2D).

We also addressed mispairing of the high-affinity mouse TCR p53 in human primary T-cells (Figure 4B). Two plasmids encoding either a wild type or functionally unresponsive scTCR p53 silCDR3α along with Cα or any complementary TCRα-chain were retrovirally transduced and enriched for gene expression in human T-cells as described before. Here, TCR Cα-mispairing of arbitrary endogenous TCRα was not detectable (MFI 4.3) as judged from expression of scTCR p53 alone. A fraction of T-cells transduced with scTCR p53 + Cα was positive for tetramer staining (CD8^+^ MFI 29.1). ScTCR p53 silCDR3α transduced along with Cα or any other unrelated TCRα-chain did not recognize the cognate antigen as expected (MFI 3.9, 4.2). In contrast, coexpression of the related TCRα p53-chain restored substantial amounts of antigen recognition (MFI 37.1), suggesting more mispairing to take place for a mouse scTCR as opposed to a human (even chimerized) one (Figure 4A).

Next, we wanted to verify the variable competitive strengths of different TCRα-chains of varying species and TCR subfamily affiliation for CD4+ and CD8+ T-cells in analogy to J-76 data (Figure 2B) but in the presence of polyclonal endogenous TCRs. In flow cytometry- and IFNγ-secretion analyses (Suppl. Figure 3A) we indeed observed the same trend: A mouse TCRα MDM2 was the most potent chain (MFIs CD4^+^ 10.5, CD8^+^ 23.6), for reasons as outlined before. In conclusion, the relative mispairing potential (i.e. the competitive strength) of a particular TCRα-chain to associate with a scTCR remains unaltered in the presence of polyclonal endogenous TCRs.

We observed some TCR Cα-mispairing for Hu Chim scTCR gp100 (Figure 4A) but not for Mu scTCR p53 (Figure 4B) in human T-cells, which we speculated to stem from a higher structural rigidity, i.e. stable folding, of a mouse scTCR-fragment (i.e. Vα-Li-Vβ). Hence, we tested this hypothesis by querying whether a higher competitive strength of any unrelated TCRα-chain, in particular a murine one, might evoke TCR Cα-mispairing (Figure 4C) as previously shown to be the case for a murinized TCRα-chain in J-76 (Suppl. Figure 2A). Expression analyses with human and mouse TCRα-chains demonstrated that mouse specimens achieved much higher scTCR p53 Vβ3-expression rates (even higher than Mu Cα (MFI 116)) than human ones (MFIs 146 and 137 *vs* 16, 13.8). The latter were in the range of the negative control scTCR p53 alone (MFI 21.7) leaving us to conclude that the human TCRα inspected here tentatively pair with a mouse scTCR. They didn't undergo neither TCRα- nor TCR Cα-mispairing. However, scTCR p53 alone exhibited a faint signal above the mock signal (MFI 4.0) that likely resulted from some interaction with any endogenous human TCRα in bulk T-cells. Consistently, only coexpression of the antigen-related TCRα p53-chain led to multimer-binding (MFI 16.1) while none of the TCRα-chains of varying competitive strengths, even TCRα MDM2 (Figure 2B, Suppl. Figure 3A), owing to their species origin or TCR subfamily affiliation were able to reconstitute tetramer binding (MFIs 2.6): Although the ‘strong’ species-related Mu TCRα MDM2 led to marked Vβ3-expression of scTCR p53 in accordance with Suppl. Figure 2B for Jurkat-76, no tetramer-staining was observed. Thus, the stable folding of a mouse scTCR allows TCRα-mispairing only with species-related but hardly ever with human (‘weak’) ones. The results of Figures 4A-4C were confirmed in IFNγ-secretion assays (scTCR p53: Suppl. Figure 3B/3C, scTCR gp100: Suppl. Figure 4).

From this we conclude that TCRα-mispairing is favored over TCR Cα-mispairing due to the larger sum of interaction forces between mouse V- and C-domains (see also explanations in paragraph to Figure 1A/1B). In general, TCR Cα-mispairing may become only prominent for strong interactions between C-domains and weaker (also transient) interactions between V-domains. TCR chains characterized by weak interactions between V- and C-domains just dissociate. Eventually, to reconcile the different behavior of human *versus* mouse scTCRs in human T-cells we propose a mechanism by which the Vα-domain of any endogenous human TCRα ‘senses’ the presence of a human or mouse Vβ-domain in a 3-domain single chain TCR to contribute to inter-chain binding which eventually determines the relative amount of TCRα- *versus* TCR Cα-mispairing, and dissociation, respectively (Suppl. Figure 5).

### Dual specificities of endogenously pp65-specific T-cells equipped with scTCR p53

Previous results showed that in human T-cells a human TCRα poorly associates with a mouse scTCR p53 (Figure 4C) and that it was hardly able to compete with Cα for binding to scTCR p53 (Suppl. Figure 3A). This in turn supports the idea that endogenous TCRβ governs binding to human TCRα as shown in J-76 (Figure 2C/Figure 2D). This should enable the generation of scTCR p53/endogenous dcTCR CMV bispecific T-cells (Figure 4D) which represent TCR-engineered T-cells of high therapeutic relevance in DLI of hematological diseases. A mechanistic proof-of-concept has been achieved for scTCR gp100/dcTCR CMV-bispecific J-76 cells (Figure 3). We retrovirally transduced 2A-linked scTCR p53/Cα or dcTCR p53 into oligoclonal pp65-specific T-cells enriched by pp65(495-503) peptide-stimulation from CMV^+^ donors [40]. Dc or scTCR p53 expression (Mu TCRβ) was prominent in the presence of the endogenous TCR pp65 (Tet pp65). Interestingly, dcTCR p53 led to a stronger decrease in multimer-staining of TCR pp65 than scTCR p53 (MFI_y_ 203 *vs* 288). Downregulation of TCR pp65 is likely mediated by TCR chain mispairing with either TCRα or TCRβ p53, or both. Obviously, the scTCR-design limited unwanted interference between both TCRs more efficiently. Moreover, we could demonstrate that scTCR p53 transduced T-cells secreted higher amounts of intracellular IFNγ than dcTCR p53 (MFIs 363 *vs* 165) after pp65 antigen encounter on T2 cells almost as much as the mock (i.e. empty vector)-transduced CMV^+^ T-cells (MFI 502). In turn, scTCR p53-transduced T-cells produced more cytokine against the cognate antigen (MFI 196) than dcTCR p53 (MFI 112) for the same reason of a limited hybrid TCR-formation. Conclusively, scTCR p53-transduced CMV^+^ T-cells were clearly bispecific for the tumor- and the virus-antigen with a substantial increase in reactivity over dcTCR p53-transduced CMV^+^ T-cells.

### Protein design of a novel artificial disulfide bond Vα-Li(Vβ) formed between a Vα- and Vβ proximal linker-resident amino acid residue

**Figure 5:**
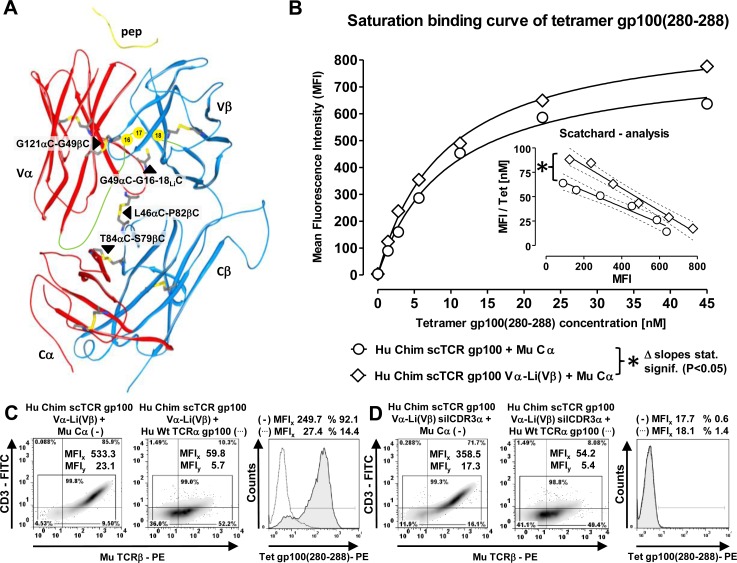
Prevention of residual mispairing by a novel artificial disulfide bond designed between the Vα-domain and the 3′-tail of the linker close to Vβ in a 3-domain scTCR **A.** Top to down side view of the human TCR crystal structure 1BD2.pdb (RCSB). 3 different disulfide bonds have been designed, G121αC-G49βC connecting Vα and Vβ, L46αC-P82βC connecting Vα and Cβ and G49αC-G16-18_Li_C connecting Vα with the carboxyterminal tail of the Gly/Ser-rich 19-mer linker at 3 consecutive positions; T84αC-S79βC supports the interaction between the autonomously expressed TCR Cα-domain and the Cβ-domain of the 3-domain scTCR (black triangles). Additionally, the 4 intradomain disulfide bonds, the recognized peptide (yellow), the hypothesized conduit of the linker (green) and positions of its glycines 16, 17, 18 (yellow circles) which were mutated to cysteines were indicated. **B.** Antigen-binding of the wild type Chim scTCR gp100 *versus* Vα-Li(Vβ) cystine-modified Chim scTCR gp100 coexpressed with Cα was quantified in dose-dependent tetramer binding saturation curves of retrovirally transduced J-76. The dissociation constant (K_d_) was calculated from half-maximal multimer binding obtained from non-linear regression analysis of the exponential saturation curve and approved for allosteric independency in a Scatchard analysis (inset) covering a broad and saturating tetramer concentration between 1 nM and 45 nM. The dashed curves illustrate narrow, non-overlapping 95% confidence intervals of the linear regression analyses reflecting a statistically significant difference of their slopes (P=0.022). For each data point 10.000 viable cells were recorded to calculate the MFI of antigen binding. **C.**/**D.** Optimized TCR gp100 constructs including the novel Vα-Li(Vβ) cystine-modified Chim scTCR gp100 were retrovirally introduced into J-76 along with either mouse Cα or Hu Wt TCRα gp100, normalized in TCR expression *via* drug-selection and analyzed for their expression (MuTCRβ) and antigen binding (tetramer) as outlined in Figure 1(C/D). Cystine-optimized Hu Chim scTCR gp100 was analyzed in (C), optimized and functionally unresponsive Hu Chim scTCR gp100 silCDR3α in (D). Assignments were as described in Figure 1(C/D). The experiment in C and D was performed twice.

Altogether, our results up to this point showed that mispairing of a single chain TCR with a full length TCRα-chain occurs on a molecular level, but is largely reduced under competitive conditions in human T-cells. In an effort to prevent so-called residual mispairing we designed different disulfide bridges by visual inspection of empirical TCR structures (mouse 1TCR.pdb, human 1BD2.pdb; RCSB [41]) in order to strengthen the interaction between the Vα- and Vβ-domain so as to eradicate any association to TCRα. Disulfide bridges are characterized by well-defined structural constraints such as clustering of the torsion angle χ_SS_ around 90° and its handedness, gauche and trans stereochemistry of χ_i_ and χ_j_, respectively, and favourable Cα-distances between 4 and 6.2 Å for the interacting cysteines [33]. We tested 3 different approaches by introducing artificial disulfide bridges into scTCR gp100 a) directly between Vα and Vβ (Vα G121C/Vβ G49C according to IMGT nomenclature), b) between Vα and Cβ (Vα L46C/Cβ P82C), and c) between Vα and the C-terminal tail of the Gly/Ser-linker [17] at three different positions (Vα G49C/Linker G16-18C) to compensate for lack of information about the folding characteristics of the 19 aa linker (Figure 5A). The side chain of Vα G49C was located in β-strand C' at the periphery of Vα and was protruding to an area where we also hypothesized the conduit of the C-terminal portion of the linker. The flexibility of the Gly/Ser-rich linker backbone was thought to appropriately allow for disulfide bond formation.

**Figure 6:**
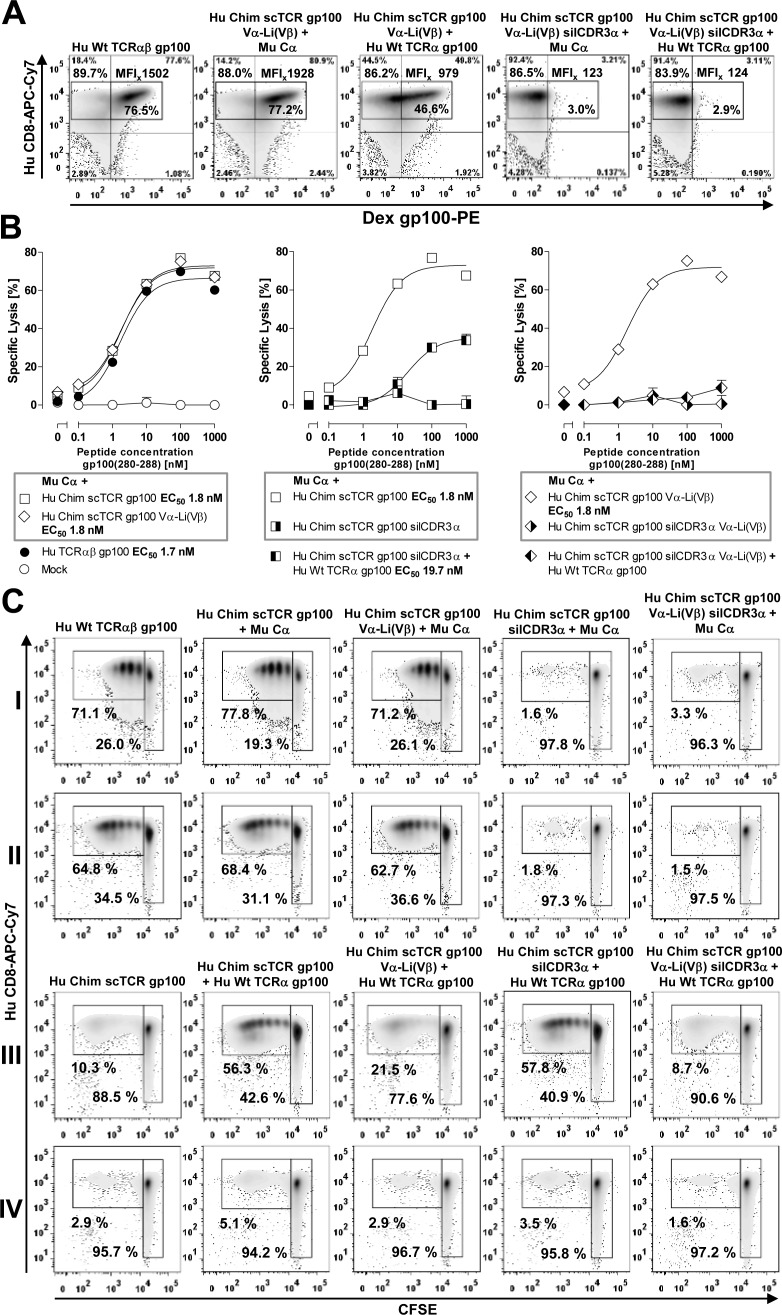
Prevention of residual mispairing in human T-cells by incorporating the Vα-Li(Vβ) disulfide bond into a human scTCR gp100 **A.** 4-10 μg of RNA encoding Cα, or TCRα gp100, or different scTCR gp100-constructs including the novel Vα-Li(Vβ) cystine-stabilized scTCR gp100 with or without the mutation silCDR3α S109Q were RNA-electroporated into MACS-purified human CD8^+^ T-cells and 20 h later analyzed for dextramer gp100(280-288)-positivity in analogy to Figure 4A. Multimer-staining was performed twice. **B.** The most relevant scTCR gp100 constructs as used in Suppl. Figures 4A and 6A were introduced into human CD8+ T-cells *via* RNA electroporation and assessed in a luciferase-based cytotoxicity assay against autologous iDCs loaded with the peptide gp100(280-288) dose-dependently for an E:T-ratio of 20:1. EC_50_-values indicate peptide concentration of half-maximal lysis. Samples were measured in triplicates. **C.** The same TCR gp100 constructs as described in Suppl. Figures 4A and 6A were electroporated into quiescent MACS-purified CD8^+^ T-cells and assessed for their ability to proliferate upon gp100(280-288) antigen encounter either presented by 10^−6^M peptide-pulsed autologous iDCs (**I/III**) or gp100 RNA-electroporated iDCs followed by endogenously processed and HLA-A2-restricted antigen presentation (**II/IV**). Beside controls, **I/II** denote responder T-cells electroporated with derivatives of Hu Chim scTCR gp100 + Mu Cα, **III/IV** responder T-cells electroporated with the same derivatives + Hu Wt TCRα gp100 in place of Mu Cα. T-cells were labeled with the proliferation dye CFSE, and after 5 days of coculture with antigen-reprogrammed iDCs at an E:T of 10:1 dilution of CFSE was quantified. Representative data of duplicates are shown. A similar result was obtained in an independent experiment.

Of all disulfide-bridges tested, only the Vα-linker engineered scTCR gp100, irrespective of the chosen Cys position in the Ser/Gly-linker, yielded a slightly better expression rate in J-76 (Figure 5C, MFI 533.3 *vs* Figure 1C, MFI 481.9) and in human T-cells (Figure 6A; MFI 1928 *vs* Figure 4A, MFI 1669) than the unmodified scTCR. This most likely resulted from the minimal effect imposed on the native TCR structure since only a single amino acid in Vα was modified while the second modification affected the artificial linker. Importantly, bridging Vα with the C-terminal proportion of the linker which is in close proximity to Vβ, resembles a direct linkage of Vα with Vβ, rendering them tightly associated and hence, was herein referred to as Vα-Li(Vβ). In an attempt to optimize the design of a mouse TCR, we chose for scTCR p53 an alternative position at Vα Q51C for cystine formation to G16C of the linker. This deals with the fact that Vα G49 in beta strand C' faces Vβ G119 of beta strand G very closely while Vα Q51 permits more space to accommodate a cystine bridge.

We quantified the structural avidities of Chim scTCR gp100 + Cα *versus* Chim scTCR gp100 Vα-Li(Vβ) + Cα to the cognate antigen by means of a tetramer saturation binding assay in flow cytometry analysis. We took advantage of J-76 cells to ensure the absence of potentially interfering endogenous TCRs (Figure 5B). Independence of cooperative effects in the dose-dependent multivalent multimer binding process was verified in Scatchard-analysis (Figure 5B, inset) giving a reasonable linearity for a broad range of tetramer concentrations. The introduction of the novel disulfide bond into Chim scTCR gp100 + Cα led to a slight increase of its avidity as read out by the lower equilibrium dissociation constant (K_d_) in non-linear regression analysis (9.1±0.7 nM) *versus* Chim scTCR gp100 + Cα engineered J-76 (9.6±1.2 nM) which corresponded to a statistically significant difference of their slopes in Scatchard-analysis (K_d_ = −1/slope, P=0.022). There is also a trend for scTCR p53 Vα-Li(Vβ) + Cα (K_d_ = 9.0±3.7 nM) *versus* scTCR p53 + Cα (K_d_ = 9.4±4.2 nM) albeit less stringent (P=0.214, data not shown).

Initial mispairing analyses of the disulfide optimized scTCR gp100 Vα-Li(Vβ) were performed in J-76 by retroviral transduction (Figure 5C/Figure 5D) in analogy to experiments outlined in Figure 1C/1D. In compliance with Scatchard-analysis, expression and antigen recognition of scTCR + Cα was somewhat lower (Figure 1C left, MFI_x_ 481.9, 239.1) than for the optimized single chain TCR (Figure 5C left, MFI_x_ 533.3, 249.7). However, this was not true for the S109Q-silenced scTCR probably due to 2 amino acid replacements impacting on its native structure (Figure 5D left, MFI 358.4 *versus* Figure 1D left, 410.6). Mispairing with Hu TCRα decreased by more than a factor of 6 (533.3/59.8; 358.4/54.2) for the optimized scTCR gp100 irrespective of being silenced in CDR3α or not (Figure 5C/5D middle) in contrast to less than a factor of 4 (481.9/125.9; 410.6/125.1) for the non-modified scTCR (Figure 1C/1D middle). Reduction in TCRβ-staining also correlated to a decrease in CD3-export to the cell surface. Importantly, this also translates to the complete extinction of residual tetramer binding (Figure 1D right, MFI 39.7) for the optimized scTCR (Figure 5D right, MFI 18.1) and IFNγ-secretion in J-76 (data not shown).

### Prevention of residual mispairing in human T-cells by incorporating the Vα-Li(Vβ) disulfide bond into a human scTCR gp100

Next, we analyzed the more relevant situation in TCR gp100 RNA-electroporated CD8^+^ human T-cells (Figure 6A) by dextramer staining correspondingly to Figure 4A. The formation of the cystine bridge led to a somewhat better expression of the optimized scTCR gp100 (Figure 6A, MFI 1928) in relation to the dcTCR gp100 (MFI 1502). However, scTCR Vα-Li(Vβ) elicited by analogy with the unmodified scTCR a modest multimer-staining most-likely resulting from TCR Cα-mispairing (46.6%, MFI 979). Mock control scTCR silCDR3α S109Q + Cα proved a background staining of about 3% (MFI 123) such as the mock control in Figure 4A (3.2%). Most importantly, optimized scTCR silCDR3α S109Q + TCRα gp100 did not exhibit any multimer positivity above background (2.9%, MFI 124) originating from TCRα-mispairing. Thus, introduction of the scTCR fragment-stabilizing disulfide bond abolished residual mispairing in terms of multimer-staining ranging around 4.2% (7.3% - ∼3.1%) (Figures 4A *vs* 6A).

We also tested this design in a peptide-titrated luciferase-based cytotoxicity-assay (Figure 6B). The scTCRs with or without the stabilizing cystine bridge Vα-Li(Vβ) proved to be as cytotoxic as the wild type dc TCR gp100 (left, EC_50_ 1.8 nM *versus* 1.7 nM). Again, residual mispairing of functionally unresponsive Hu Chim scTCR gp100 silCDR3α with wild type TCRα gp100 could be observed for moderate to high peptide pulses (middle, 10-1000 nM; EC_50_ 19.7 nM). Incorporation of the cystine bridge prevented almost entirely the formation of hybrid scTCR gp100/TCRα gp100 (right). This was largely confirmed by functional data from a peptide-titrated IFNγ-secretion assay (Suppl. Figure 4) and a proliferation assay based on peptide loading (Figure 6C I/III). Notably, the disulfide bond stabilized scTCR was able to equally recognize an A2^+^/gp100^+^ human melanoma cell line (data not shown) as experimentally demonstrated in more detail for the unmodified Hu Chim scTCR gp100 elsewhere [17].

### Residual mispairing is less prominent when taking into account endogenous antigen processing and could be prevented by incorporation of a novel cystine bridge

We also aimed at testing residual mispairing of TCR gp100 RNA-electroporated human quiescent CD8^+^ T-cells in a CFSE-based proliferation assay against either gp100 peptide loaded immature dendritic cells (Figure 6C, rows I/III) or full length gp100 antigen electroporated iDCs (rows II/IV) for the same panel of TCR gp100-engineered cells such as used in Figures 4/6A. RNA electroporation of the full length gp100 antigen is supposed to represent a more physiologic situation than a saturating synthetic peptide pulse with respect to antigen quality, not quantity (due to the vast excess of electroporated RNA), because the antigenic protein is submitted to endogenous processing by the proteasome for e.g. HLA-A2-restricted peptide presentation [28] which may affect duration and mode of antigen presentation. Moreover, the longer period of time to mount a progressive stimulation of T-cells may account for a higher sensitivity of this assay: On average, proliferation upon antigen encounter gave rise to 4-5 distinct daughter populations peaking between populations 2-4 after 5 days of coculture.

The modest dextramer positivity observed for Chim scTCR gp100 in Figure 4A could be confirmed by a low proportion of proliferating cells towards peptide loaded targets (III, 10.3 %) most-likely due to some TCR Cα-mispairing with any endogenous TCRα. In case of antigen processing the frequency of proliferating T-cells (IV, 2.9%) dropped down to almost background, which was defined here by scTCRgp100 silCDR3α + Cα (II, 1.8%). Noteworthy, proliferation of scTCR gp100 + Wt TCRα gp100-modified T-cells decreased from 56.3% for peptide-pulsed targets to 5.1% for processed antigen-presenting iDCs which may still account for both, TCR Cα- and TCRα-mispairing. Hence, we hypothesize, that the amount of mispairing as it is read out for particularly high peptide loads (i.e. 10^−6^M) in standard operation protocols tends to be overestimated.

All TCR gp100-modified T-cells being regarded as strong responders demonstrated almost equal and high proliferation rates for both, peptide loaded and antigen-processing iDCs (row I/II, dcTCR gp100, 71.1%/64.8%, scTCR gp100 + Cα, 77.8%/68.4%, scTCR Vα-Li(Vβ) + Cα, 71.2%/62.7%). Elevated amounts of mispairing with TCRα gp100 were observed only for those scTCR constructs which have not been optimized by a disulfide bond (III, scTCR + TCRα gp100, 56.3%; scTCR gp100 sil CDR3α + TCRα gp100, 57.8 %). Introduction of the cystine-bridge reduced mispairing down to 21.5% for scTCR gp100 Vα-Li(Vβ) and 8.7% for scTCR gp100 Vα-Li(Vβ) silCDR3α, respectively, for gp100 peptide-pulsed cells (III). However, this corresponded not to background defined here by scTCRgp100 silCDR3α + Cα (I, 1.6%). In case of antigen-processing (IV) hybrid TCR-formation in those responder cells declined to 2.9% and 1.6%, respectively, the latter one which, most importantly, equals background staining (I/II): The lower percentages for the CDR3α-silenced *versus* the functionally responsive ones (i.e. IV, 3.5 *vs* 5.1%) and also for the panel of cystine-un/modified (i.e. III, 8.7 *vs* 21.5%; IV, 1.6% *vs* 2.9%) scTCR constructs could be assigned to TCRα-mispairing alone *versus* TCRα- + TCR Cα-mispairing in line with conclusions drawn from dextramer stainings (Figures 4A/6A). In summary, true residual TCRα mispairing which took place in the range of 1.7% (i.e. IV-II, 3.5%-1.8%) was eliminated by introducing such kind of a stabilizing Vα-Li(Vβ) disulfide bond (i.e. IV, 1.6%) in an experimental setting of endogenous antigen presentation.

### Residual mispairing could not be avoided by 2A-linkage of mouse scTCR p53/Cα in human T-cells but by incorporating the novel disulfide bond

**Figure 7:**
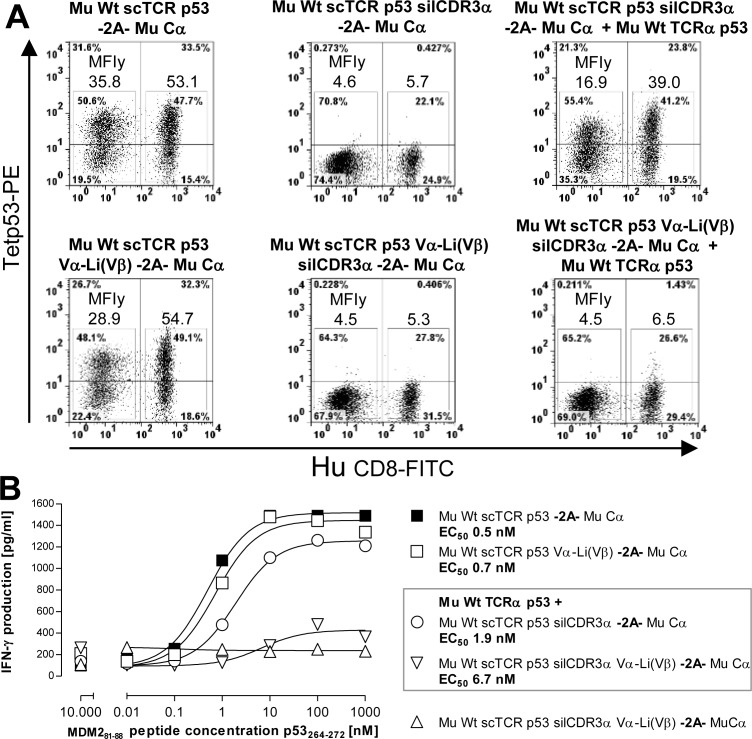
Reduction of residual mispairing in human T-cells by incorporating the novel disulfide bond into a mouse scTCR p53 **A.** Different Mu Cα 2A-linked scTCR p53 constructs, either unmodified or stabilized by Vα-Li(Vβ) (Q51C-G16C), with or without the silencing mutation silCDR3α D109A, were either expressed alone or along with Mu Wt TCRα p53 and assessed in p53(264-272)-specific multimer-binding. Mu Cα 2A-linked Mu Wt scTCR p53 silCDR3α w/wo Vα-Li(Vβ) served as background controls. **B.** The same scTCR p53 constructs were also assessed in an IFNγ-secretion ELISA after coculture with peptide-pulsed T2 cells dose-dependently at an E(Vβ3^+^CD8^+^):T-ratio of 0.1:1. Here, Mu Wt scTCR p53 silCDR3α Vα-Li(Vβ) -2A- Mu Cα served as background control. Data are shown as mean of duplicates.

Subsequently, we also assessed whether a related Vα-Li(Vβ) disulfide bond in scTCR p53 achieved a similar favourable effect on residual mispairing in a retroviral vector system encoding scTCR p53-F2A-Mu Cα on a single plasmid (Figure 7). Meanwhile, 2A-constructs were routinely used in the clinic to introduce cancer-specific dcTCRs into human T-cells [42]. The genetic linkage of Mu Cα *via* 2A to the scTCR p53 was thought to kinetically favor interaction of the latter with Cα in cis over any endogenous TCRα provided in trans in a cell during protein biosynthesis and thus, to prevent mispairing. TCR p53 is a CD8-independent high affinity TCR originating from A2-transgenic mice and hence, stains CD8+ and CD4+ T-cells [31]. However, in flow cytometry analysis (Figure 7A) we still observed considerable mispairing with TCRα (MFI, CD4+ 16.9; CD8+ 39.0) with reference to scTCR p53 + Mu Cα (MFI, CD4+ 35.8; CD8+ 53.1). Introduction of the cystine modification Vα-Li(Vβ) into scTCR p53 was able to largely reduce residual mispairing (MFI, CD4+ 4.5; CD8+ 6.5). The same TCR p53 constructs have been tested in a peptide-titrated IFNγ-ELISA (Figure 7B). Efficient IFNγ-secretion was observed for the transduced T-cells which were equipped with the wild type or disulfide-optimized p53(264-272)-specific scTCR-2A-Cα giving rise to IFNγ levels of more than 1400 pg/ml at saturating peptide loads (i.e. >10 nM). Remarkably, unmodified scTCR p53 silCDR3α was prone to profound mispairing with murine TCRα p53 resulting in IFNγ levels of more than 1200 pg/ml. Contrary, the cystine-optimized scTCR p53 silCDR3α D109A, 2A-linked to Mu Cα and co-transduced with the same TCRα p53, exhibited a sizable decrease, but not extinction, of IFNγ-secretion down to less than 400 pg/ml. These results corresponded to the multimer-stainings as shown in Figure 7A.

Surprisingly, the amount of mispairing was even more pronounced for a mouse TCRα p53 competitor than a human TCRα gp100 one based on independent coexpression as demonstrated in Figure 6A/6B or Suppl. Figure 4. Retrospectively, this most-likely resulted from a stronger Mu Cα/Mu Cβ- than Hu Cα/Mu Cβ-interaction in those hybrid TCRα/scTCRs [5, 8]. It is tempting to speculate, that the elimination of residual mispairing would have been even more impressive when a human and hence, less competitive TCRα p53-chain as a ‘surrogate’ for any endogenous one would have become available. Actually, the competition with a human TCRα represents the physiologic situation. In summary, even in a 2A-linked retroviral vector construct, which should kinetically assist chain pairing of the nascent polypeptides, a TCRα is able to compete with Mu Cα for binding to a scTCR. Therefore, a strategy such as a disulfide bond modification may also be required for such a clinically relevant vector design.

### The novel Vα-Li(Vβ) disulfide bond improved the functional and structural avidity of a fragile scTCR pp65

Finally, we addressed the question whether such a cystine-modification may not only affect chain pairing but may also help to stabilize fragile scTCRs characterized by poor antigen recognition due to dissociation or unfolding of their Vα/Vβ-domains [43]. We analyzed cytotoxicity of an optimized Chim scTCR pp65 Vα-Li(Vβ) + Cα compared with an unmodified scTCR pp65 generated from a human dcTCR [27] in a retroviral vector system in human T-cells (Suppl. Figure 6A). TCRβ-expression of the cystine-modified scTCR pp65 was slightly better than for the unmodified one (data not shown). Two independently isolated bacterial clones encoding the optimized scTCR pp65 were more efficient in killing pp65(495-503) peptide-titrated T2 target cells (EC_50_ 0.09 nM) than the unmodified scTCR pp65 (EC_50_ 0.33 nM). However, lysis efficacy of the optimally designed dcTCR (EC_50_ 0.03 nM), modified by murinization of its C-domains [5] and linkage of TCRα and TCRβ *via* the self-processing peptide 2A [37], was not fully achieved. This might demand to consider the combination with other well-known optimization strategies [43], although actually, non-optimized Hu Wt TCRαβ pp65 instead of Hu Chim TCRαβ pp65 2A would have been the more appropriate reference if available. Of note, the cystine modification Vα-Li(Vβ) triggered multimer-positivity of 2A-linked Chim scTCR pp65/Mu Cα after 2 rounds of peptide restimulation (Suppl. Figure 6B) which underlines its pivotal role in triggering proper protein folding.

## DISCUSSION

Adoptive transfer of TCR-engineered tumor reactive T-cells represents a clinically effective therapeutic strategy [1] that may allow overcoming the lack of high affinity T-cells specific for self tumor associated antigens due to central and peripheral tolerance mechanisms [44]. However, this approach may have important limitations arising from the mispairing with endogenous TCRs. In this report, we assess the extent of hybrid TCR-formation for a single chain TCR format and identify specific modifications to those TCRs that reduce mispairing to negligible levels.

First clinical trials prove the successful eradication of metastatic tumors such as melanoma in some patients [42]. Recent experiments and trials also uncovered that TCRs were able to recognize the cognate antigen in normal tissue (on-target/off-tumor) or even may cross-react with related or unrelated antigen (off-target/off-tumor), which eventually may end up with mild to severe autoimmune GvHD-like symptoms [1]. Additionally, mispairing of introduced TCR-chains with endogenous ones may misguide T-cells to unforeseen immune attacks against self [2]. However, in none of the finalized clinical trials till 2010 overt autoimmune reactions due to hybrid TCR-formation have been proven [45]. Contrary, in an OT1-TCR-based syngeneic mouse model GvHD-like symptoms [3], and in a panel of human phenotyped LCLs tested against TCR-engineered human T-cells neo-reactivities attributable to TCR-mispairing have been observed [4].

A cellular approach to reduce the risk of mispairing is the dual immunoreceptor-strategy in donor lymphocyte infusions (DLI) of engineered CMV/HA-2-bispecific T-cells after bone marrow transplantation of relapsed or refractory leukemia CMV^+^ patients [32, 46]. The vast amount of viral antigens multiplies the activation of HA-2-engineered T-cells while the endogenous CMV-specific TCR (oligo)clonality confines the number of neoantigens originating from mispairing with the introduced anti-leukemic TCR. Here, we were able to demonstrate in a proof-of-concept for a novel single chain format, that indeed a scTCR of the gp100(280-288)- or p53(264-272)-specificity was independently expressed and operative in the presence of a dcTCR of the CMV pp65(495-503)-specificity. Since p53 is overexpressed in many leukemias, this strategy yielded scTCR/dcTCR bispecific T-cells valuable for DLI in hematopoietic diseases. We also designed a CMV pp65-specific scTCR, which required stabilization by a novel disulfide bond as discussed later. The latter, either in a dc/sc- or in a even more mutually exclusive sc/sc-configuration in bispecific T-cells, may be used in those cases where CMV-specific T-cells could not be isolated from these patients.

For this, we took advantage of the coexpression of a in Cβ murinized (or chimerized) 3-domain Vα-Vβ-Cβ single chain TCR, which was hypothesized to avoid mispairing due to sterical hindrance, along with an autonomously expressed murine TCR Cα-domain to still allow for assembly of the native TCR/CD3-complex (Suppl. Figure 1D; [17]) as opposed to alternative strategies [13]. This process is mediated by charged residues in their transmembrane regions among those Cα recruits CD3ζ_2_ and CD3δε as the most relevant ones [19]. Experiments in retrogenic mice revealed their importance for ITAM-dependent scalable T-cell signaling to preserve peripheral T-cell homeostasis [47]. The functional sidedness of the TCRαβ framework offers a mechanism by which only high antigen densities trigger dimerization of TCR/CD3 complexes to yield sustained T-cell signaling while low antigen densities give rise to only transient signals [48, 49]. Dimerization is supposed to be driven by the unusual top β-strands of Cα which again emphasizes the pivotal role of this domain in T-cell signaling. Thus, we hypothesize that titration of TCR reacticvity may also serve as a safeguard for specificity to prematurely abrogate T-cell signaling in case of ‘uncertainties’ about the self/non-self nature of low-level antigens in contrast to the plain non-self origin of e.g. high-level viral antigens.

The limiting native CD3 complex does not necessarily represent a fundamental drawback of this approach since it is not the amount but predominantly the affinity of the introduced TCR that confers anti-tumor reactivity [9, 50]. Additionally, xenoreactivity against mouse C-domains may be overcome by minimal murinization to a few amino acid residues which are not accessible to the immune system because they are buried at their interface [20, 21]. Secondly, human anti-mouse antibody reactions against mouse TCR p53 in a clinical study were confined to TCR V-domains, and not directed against their C-domains [51].

However, Aggen et al. observed elevated amounts of mispairing of such a 3-domain scTCR with TCRα in non-primary T-cell lines such as a murine T-cell hybridoma 58α^−^β^−^ lacking endogenous TCRs [16, 52]. Indeed, we also reported on mispairing in a human Leukemia cell line Jurkat-76 (J-76) devoid of interfering endogenous TCRs (ASGCT annual meeting 2011, Seattle, USA, abstract #221 in Mol Ther Volume 19, issue suppl. 1). This unfavourable reaction has been confirmed here for 2 scTCRs specific for gp100(280-288) and p53(264-272) of 2 different species origins, and additionally, in 2 different expression systems.

From previous studies on the folding stability of antibody scFv-fragments it became evident that antigen recognition is compromised by either dissociation of their V-domains due to poor interaction at their interface or by unfolding of either domain due to weak intrinsic stability [36]. These mechanisms are believed to apply also to scTCR-fragments to even higher amounts originating from a weaker folding stability of membrane resident TCRs which is largely governed by the TCR-subfamily affiliation [43, 53]. This weak point facilitates mispairing of a 3-domain scTCR with an endogenous TCRα-chain.

Most importantly, in human T-cells mispairing of human Chim scTCR gp100 with full length human TCRα declined to a large degree but was still detectable. We reasoned that the much more complicated, namely TCR chain-competitive, situation in human T-cells may have resulted in a different outcome compared to the data collected in 58α^−^β^−^ or J-76: The presence of endogenous TCRβ (beside endogenous TCRα) was supposed to mitigate the amount of hybrid TCRα/scTCR-formation. Moreover, we hypothesized that the presence of a (1-domain) mouse Cα successfully competes with the weak interspecies interaction of Cα of a (2-domain) human TCR for binding to murine Cβ of the (chimerized 3-domain) human scTCR. This is in line with results for a mouse scTCR p53, where TCRα-mispairing was much more pronounced when a more competitive mouse TCRα as opposed to a human one has been used instead. Even the immediate linkage of a mouse scTCR p53 to mouse Cα on a single genetic construct by means of a self-processing peptide 2A which was believed to favor heterodimer formation of their nascent polypeptides [37] did not prevent mispairing with mouse TCRα p53. This contradicts to the general view that the usage of 2A-linked dcTCR constructs represents an efficient method to provide not only optimal (i.e. stoichiometric) expression but also exclusive chain pairing of the designated ones. However, the whole set of results supported the idea that mispairing of a mouse scTCR with full length human TCRα is very low. This is of importance since meanwhile in a number of clinical trials high-affinity TCRs derived from A2-transgenic mice were used [42]. Nevertheless, a few human TCRα-chains were still able to compete somewhat which is consistent with previous conclusions drawn from the empirical evidence of interspecies hybrid dcTCRs [8]. Of note, a titratable competition of TCRα-chains carrying identical TCR Cα-domains pinpoints to the dependency of TCRα/scTCR-chain pairing on the interaction forces between their TCR subfamily-affiliated V-domains and also possibly between their clonotypic CDR3α/β loops as demonstrated recently for dcTCRs [24]. Conclusively, our results strongly suggest that mispairing of a human (chimerized) scTCR with human (endogenous) TCRα hardly occurs and even less for a murine scTCR.

To unravel the mechanisms by which mispairing was reduced in human T-cells, we set up a panel of coelectroporated TCR-chains in J-76 to mimic the TCR competitive situation in human T-cells. Initially, we assessed the competitive strength of TCRα *vs* Cα for binding to human scTCR gp100. Although in scTCR/Cα interaction was confined to Cα and Cβ, it could be detected to substantial amounts in particular against TCRα competitors of human origin. Hence, strategies, that have successfully been applied to dcTCRs to enhance chain pairing, i.e. the murinization of the C-domains [5] and the introduction of an artificial disulfide bond [6] compensate the lack of V-domain interaction and foster successful competition of Cα. In TCRβ *vs* scTCR gp100 competition experiments both, a ‘weak’ TCRα pp65 or ‘strong’ TCRα gp100-chain are prone to interact with either their thymically coevolved or an unrelated TCRβ-chain. This supports the notion that selfpairing to a dcTCR, even a weak one [24], is the superior reaction to mispairing with a 3-domain scTCR and, beside the aforementioned competitive strength of optimized Cα, helps to explain why scTCR/Hu TCRα-mispairing is scarcely observed in T-cells.

We also addressed the question whether Cα alone of any endogenous TCRα is able to reconstitute the predefined antigen-specificity of a 3-domain scTCR (i.e. TCR Cα-mispairing) or full length TCRα-chain pairing solely takes place and reprograms antigen-specificity of the resulting hybrid scTCR/TCRα (i.e. TCRα-mispairing). To our best knowledge the former reaction has not experimentally been ascertained before to take place. Partial or temporary unfolding or dissociation of Vα by analogy to scFv's [36], and/or domain rearrangements in TCRα may explain its incidence. In J-76, we noticed some TCR Cα-mispairing for a strongly competing human TCRα gp100 and not for a ‘weak’ human as opposed to a ‘stronger’, in Cα murinized TCRα pp65. However, we did not observe this side reaction for a mouse scTCR p53, but more likely full length TCRα-mispairing with murine TCRα, and dissociation of human TCRα, respectively. Hence, we hypothesize a mechanism by which the Vα-domain of any human (endogenous) TCRα ‘senses’ the presence of a human or mouse Vβ-domain in a given 3-domain single chain TCR which implies at least transient access of Vα(TCRα) to Vβ(scTCR) resulting from V-domain dissociation and/or partial unfolding (Suppl. Figure 5). For the same reason, TCR Cα-mispairing with a human, but not mouse scTCR was also observed in human T-cells. We suggest, that TCR Cα-mispairing provides a supportive effect in gene therapy since antigen-specificity is preserved in marked contrast to the counter-productive effect of TCRα-mispairing.

In a CFSE-based proliferation assay we observed mispairing to occur to much less amounts when immature DCs were electroporated with the full length antigen gp100 while recognition by the most potent TCR gp100-engineered T-cells was preserved. Hence, endogenous processing of full length antigen by the proteasome might reflect a more physiologic situation in terms of antigen quality such as duration [28] or mode of antigen presentation. From this we conclude that peptide-loading of any APC, which still constitutes the most commonly applied method to date, might lead to an overestimate of true antigen recognition, here originating from mispairing of any TCRα with a scTCR.

However, even a frequency of as low as 1.7% of antigen-responsive proliferating T-cells may pose a risk to develop GvHD-like symptoms *in vivo*. Protein design exploited the introduction of scTCR fragment-stabilizing pairs of interacting amino acid residues centered at the V-domains' interface deduced from either highly stable antibody scFv-fragments (Vα-P^50^-Vβ-L^50^) [43, 53] or TCR crystal structures (Vα-Q^44^-Vβ-Q^44^) [23]. In our hands, these amino acid replacements led indeed to an improved expression of a weakly stable tight junction protein-specific 3-domain scTCR but largely abolished antigen recognition. Hence, we decided to engineer novel disulfide bonds between V-domains by analogy to a recently described cystine bridge in TCR C-domains [6, 54]. We were able to slightly improve structural and functional avidity of especially human scTCRs but most importantly, to eliminate residual mispairing by modeling an artificial disulfide bond between Vα and the C-terminal tail of the artificial linker close to Vβ. This leaves the framework residues of the wild type scTCR as much as possible unchanged so as not to compromise structure and function. A similar approach has been utilized to generate a more stable soluble peptide-β2m-HLA-A2 trimer by immobilizing the peptide into the HLA peptide binding groove *via* a so-called ‘disulfide trap’ [55]. Of note, a weakly stable scTCR of CMV-specificity could be enhanced in function almost 4-fold. This novel approach is ideally suited to not only prevent mispairing but also to stabilize fragile scTCR-fragments which is a common characteristic of engineered TCRs [43].

Proteolysis of linker-attached Vα might also explain the association of endogenous TCRα to scTCR during assembly in the endoplasmic reticulum of a T-cell. Actually, western blot analysis in non-reducing SDS-PAGE detected a very faint 45 kD-band which might originate from a proteolysed disulfide-bonded Vβ-Cβ-S-S-Cα-fragment (supplemental in [17]). However, i) the largely reduced onset of scTCR mispairing in a TCR competitive environment such as J-76 endowed with a dcTCR and ii) the ample prevention of residual mispairing by an artificial disulfide bond strongly supports the notion that Vα is still covalently attached to the single chain TCR-fragment.

Here, we could demonstrate that mispairing of a 3-domain scTCR with any TCRα is largely reduced in human T-cells in contrast to artificial cell lines such as 58α^−^β^−^ or J-76 lacking endogenous TCRs. Residual mispairing could be eliminated by improving scTCR V-domain pairing through a novel artificial disulfide bond. This approach may be applicable to the generation of bispecific T-cells for donor lymphocyte infusions. Beyond that, optimized 3-domain scTCRs permits the presence of polyclonal endogenous TCRs so as to reconcile CD3 complex-dependent physiologic T-cell signaling with the prevention of hybrid TCR formation for the sake of definite on-target-specificity.

## MATERIALS AND METHODS

### Peptides, antibodies, and multimeric pA2.1 complexes

Peptides p53(264-272), gp100(280-288), MDM2(81-88), and pp65(495-503) were synthesized by Biosyntan (Berlin) or PSL (Heidelberg, GER). The monoclonal antibodies (mAbs) used were against Hu CD4-, or CD8-FITC, Hu Pan TCRαβ-, Hu Vβ13.1-, Vβ14-PE (Beckman-Coulter, Krefeld), Hu CD3(cl. HIT3a)-FITC, CD8-APC-Cy7, Mu TCRβ-, Vβ3-PE, Hu IFNγ-APC (BD Biosciences, Heidelberg, GER). PE- or PE-Cy7-labeled tetrameric p53-specific or gp100 pA2.1 complexes were obtained from LICR (Epalinges, Switzerland), pp65(495-503) tetramers were from Beckman-Coulter, gp100(280-288)-specific dextramers from Immudex (Copenhagen, Denmark).

### Plasmids, TCR cloning

For RNA-electroporation, genes encoding gp100 [30]-, CMV IE1(316-324)- or pp65 [24]-specific TCRα and TCRβ were either cloned into pGEM4Z vector [34] *via* XbaI/XhoI, or a modified pST1 vector [28] *via* SmaI-SfoI/BamHI. For retroviral transfer, genes encoding a gp100-[30], p53 [31]-, MDM2 [56]-, CMV pp65 [27, 40]-, or AML-specific TCR [38] were cloned into a modified pBullet vector [8] *via* NcoI/BamHI or pMP71 [27]. Restriction enzymes were from NEB (Frankfurt, GER). Bacterial amplification in XL1-Blue, Top10, or JM109 and purification of plasmids were done according to manufacturer's instructions (Agilent technologies, Waldbronn; Life technologies, Darmstadt; NEB, Frankfurt; Qiagen, Hilden, GER). Amino acid residue replacements in TCR coding sequences were conducted following the protocol of the “Quikchange Site Directed mutagenesis”-kit (Agilent technologies). A chimerized 3-domain scTCR pp65 was generated from a 2A-linked chimerized dcTCRαβ pp65 [27] by overlap extension PCR. The full length antigen gp100 was isolated *via* RT-PCR from a gp100^+^ melanoma cell line and cloned into pST1. All final plasmids encoding receptors and antigen gp100 were thoroughly sequenced (Genterprise, Mainz MWG-Eurofins, Ebersberg, GER).

### PBMCs and cell lines

Bulk CD4^+^CD8^+^ T-cells were obtained from buffy coats of Hu A2.1^+^ donor PBMC. For this, mononuclear cells were separated by Ficoll (Biochrom) gradient centrifugation and cryopreserved. Pure CD8^+^ T-cells were isolated from PBMCs by magnetic CD8 Microbeads (Miltenyi-Biotec, Bergisch-Gladbach, GER) selection, pure immature dendritic cells were obtained from PBMCs by magnetic CD14 Microbeads selection, subsequently differentiated to iDCs by the addition of GM-CSF (f.c. 1000 U/ml) and IL-4 (f.c. 1000 U/ml, Miltenyi-Biotec) for 4 days. Human T-cells and differentiated PBMCs were cultivated in RPMI supplemented with 10% heat-inactivated human AB serum and 2mM (1x) glutamine. The amphotropic packaging cell lines Phoenix-Ampho were obtained from American Type Culture Collection (ATCC/LGC, Wesel, GER). They were grown in HEPES-buffered (25 mM) DMEM supplemented with 1x glutamine, 1x penicillin-streptomycin, 1x nonessential amino acids, and 10% (v/v) FCS (Cambrex, Wiesbaden, GER). T2 is a human HLA-A2^+^ TAP-deficient lymphoblastoid cell line; K562-A2 is a human chronic myelogenous leukemia cell line transfected with HLA-A2, Jurkat clone 76 is a human T-cell leukemia deficient in TCRα and TCRβ expression [26]. All cell lines were maintained in RPMI 1640, 10% heat-inactivated FCS, 2 mM glutamine, 50 μM geneticin or 1x penicillin/streptomycin. The generation of oligoclonal CMV pp65(495-503)-specific T-cells were performed according to [40].

### *In vitro* transcription of TCR or gp100 RNA

IVT TCR RNA was generated by 2 different protocols: the generation of RNA for experiments in J-76 was as previously described [34] for a pGEM4Z-based RNA transcription vector. Alternatively, the generation of RNA for experiments in human CD8^+^ T-cells and iDCs were basically performed as described in [28] for a pST1-based RNA transcription vector. Integrity of RNA was checked by photometric analysis at 260/280 nm and a 1.5% RNA formaldehyde/MOPS-gel.

### RNA electroporation of Jurkat-76, CD8^+^ T-cells or iDCs, retroviral transduction of bulk T-cells

5 × 10^6^ J-76 cells were washed and resuspended in OptiMEM (Invitrogen) at 25 × 10^6^ cells/mL. Electroporation was performed with the GenePulser Xcell system (Bio-Rad, Munich, GER) applying a square wave pulse of 400V, 5 ms, to 5 × 10^6^ cells with 5μg RNA derived from pGEM4Z. Alternatively, up to 20 ug RNA derived from pST1 were electroporated into T-cells with a CytoPulse-PA-4000 device (BTX Harvard Apparatus, Holliston, MA, USA) applying a single pulse at 495V, 10 ms, or into iDCs at 300V, 12 ms. Retroviral transduction of bulk CD4^+^/CD8^+^ T-cells and expansion with CD3/CD28-beads (Life Technologies) were performed as described previously [8]. T-cells transduced with 2 plasmids encoding either TCR-chain were normalized for TCR expression by puromycin and neomycin drug-selection, T-cells transduced with F2A-linked constructs [37] were selected only by puromycin. Retroviral transduction and peptide-specific expansion of TCR-engineered T-cells in the backbone of pMP71 was performed as described in [27]: Briefly, one round of restimulation of T-cells (0.5×10^5^) was achieved by coculture with irradiated feeder PBMCs (2×10^6^) and 10^−4^M peptide-loaded T2 (2×10^5^) for 10 days.

### Functional assays and EC_50_-calculations

IFN-γ ELISPOT assays of RNA electroporated J-76 and human T-cells against T2 and K562-A2, respectively, were performed in duplicate wells for 20h [34, 57]. Proliferation of RNA-electroporated and CFSE-labeled (0.8 μM of 5(6)-CFDA, Life Technologies/Molecular Probes) quiescent CD8^+^ T-cells upon encounter with gp100(280-288)-loaded or full length gp100-electroporated iDCs were quantified by CFSE-dilution after 5 days of coculture for parental and daughter populations in flow cytometry on a CANTO II-HTS device in duplicates (Becton-Dickinson). IFNγ-ELISAs of TCR-engineered human T-cells against T2 or K562-A2 were conducted with the “Human IFNγ OptEIA ELISA Set” (BD, Heidelberg)-, or the “Human IFNγ ELISA Ready-SET-Go” (eBioscience, Frankfurt)-kit according to the manufacturer's protocol. A luciferase based cytotoxicity assay was performed as described elsewhere in detail (Omokoko et al., J. Immunol. Res., 2016, doi: 10.1155/2016/9540975). Briefly, immature DCs were electroporated with luciferase IVT RNA and after 20h loaded with gp100 peptide (280-288) dose-dependently. 1 × 10^4^ peptide loaded targets were cocultured with 2 × 10^5^ TCR RNA electroporated CD8^+^ T-cells in a 96 well plate. After 3 h D-luciferin (BD Biosciences) was added to each well. After 4 hours intracellular luciferase activity was assessed using a Tecan Infinite M200 reader. Lysis efficacy is given as percentage to the difference of minimal lysis defined by target cells only and maximal lysis defined by complete lysis of target cells with Triton X-100. EC_50_ as a measure of the ligand concentration yielding half-maximal IFNγ-secretion (ELISA) or lysis was calculated from peptide titration experiments and dose-response sigmoidal regression analysis according to *Y* = Top/(1 + 10^((logEC_50_-*X*))) with GraphPad Prism v5.0 (GraphPad Software Inc, San Diego, CA, USA).

### Flow cytometry and Scatchard-analysis

TCR expression was determined in flow cytometric analysis on FACS Calibur- or FACS Canto-, proliferation on HTS plugged to Canto II (BD Biosciences)-devices. Forward/sideward scatter was determined to gate on viable cells. Intracellular IFNγ-staining was performed according to a standard protocol of Becton-Dickinson including ‘GolgiStop’-solution and ‘Perm/Wash’-buffer I. Antigen-binding was quantified in dose-dependent tetramer binding saturation curves of retrovirally transduced J-76 strictly controlled by time of incubation (20 min at RT), assay volume (200 μl), and cell density (1×10^5^) for each sample. The dissociation constant (K_d_) was calculated from half-maximal multimer binding obtained from non-linear regression analysis of the exponential dose-escalating saturation curve according to MFI=B_max_*[Tet]/(K_d_ + [Tet]) and assessed for allosteric independency in a Scatchard analysis, from which the K_d_ could be recapitulated as the negative inverse of its slope (K_d_ = −1/slope). Analyses were performed with Graphpad Prism v5.0.

### Nomenclature and graphic display of TCR structures and modeling

Nomenclature of TCR primary structures for variable domains was essentially as described in Lefranc et al. [35] and implemented in the IMGT database (IMGT^®^, the international ImMunoGeneTics information system^®^, http://www.imgt.org/, [58]): Mu TCR p53 was assigned to TRAV9D-4 and TRBV26, Hu TCR gp100 to TRAV13-1 and TRBV27, Hu TCRs CMV pp65 to TRAV24 and TRBV6-5; Mu TCR MDM2 to TRAV13D-4 and TRBV19, Hu TCR AML to TRAV39 and TRBV19, Hu TCR CMV IE1 to TRAV22 and TRBV7-8.

The crystal structure atom coordinates of a Mu H-2K^b^-restricted TCR, 1TCR [23], and a Hu HLA-A*0201-restricted TCR, 1BD2 [59], were downloaded from the Research Collaboratory for Structural Bioinformatics (RCSB) protein data bank [41] and visualized and analyzed with Swiss-PdbViewer version 3.7 [60]; amino acids such as cysteines/cystines were designed by exploiting the built-in option to browse a knowledge-based rotamer library and by adjusting the side chain dihedral angles through all empirical conformers. Atom distances, angles, and H-bond calculations have been computed and manually verified so as not to violate protein structure constraints. For graphic illustration, solid three-dimensionally rendered protein models have been compiled in POV-Ray v.3.1 (www.povray.org/povlegal.html).

## SUPPLEMENTARY MATERIAL FIGURES



## Abbreviations

A2: serotype of HLA
AML: acute myelogenous leukemia
APC: antigen presenting cell
CAR: chimeric antigen receptor
Cα: TCRα C-domain
CDR3α: complementarity determining region of loop 3 of TCRα
Chim: chimerized
c.l.: cell line
CMV: cytomegalovirus
dc: double chain
DC: dendritic cell
Dex: dextramer
DLI: donor lymphocyte infusion
EBV: Epstein-Barr virus
GER: Germany
sil: silenced
GvHD: Graft *versus* Host Disease
Hu: human
iDC: immature DC, IVT, *in vitro* transcription
J-76: Jurkat-76
Li: Glycine/Serine-rich 19-amino acid linker
MFI: mean fluorescence intensity
Mu: murine
sc: single chain
sil: silenced
TAA: tumor associated antigen
TCR: T-cell receptor
TCRα: TCR alpha-chain
TCRβ: TCR beta-chain
TCRαβ: TCR alpha/beta-chains
Tet: tetramer
TIL: tumor infiltrating lymphocyte
vs: *versus*
