# Supplementary material for: An optimized single chain TCR scaffold relying on the assembly with the native CD3-complex prevents residual mispairing with endogenous TCRs in human T-cells
Source: Oncotarget. 2016 Mar 26;7(16):21199–221. doi: 10.18632/oncotarget.8385 (PMC5008279; doi:10.18632/oncotarget.8385)
Supplement: Supplementary file 1 [file oncotarget-07-21199-s001.pdf]

# An optimized single chain TCR scaffold relying on the assembly with the native CD3-complex prevents residual mispairing with endogenous TCRs in human T-cells

**Supplementary Material**

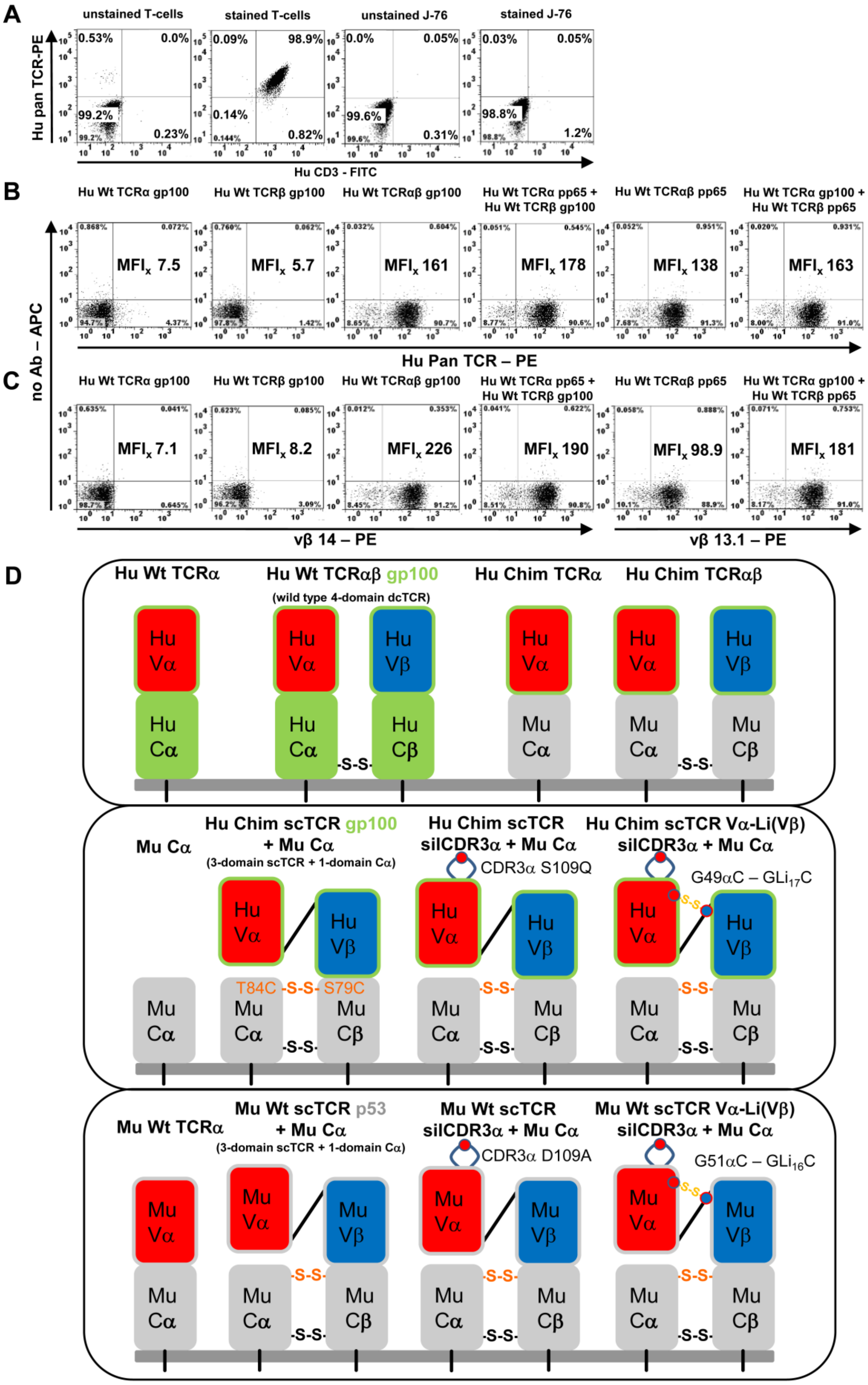

A
unstained T-cells
stained T-cells
unstained J-76
stained J-76
Hu pan TCR-PE
Hu CD3 - FITC
B
Hu Wt TCRα gp100
Hu Wt TCRβ gp100
Hu Wt TCRαβ gp100
Hu Wt TCRα pp65 + Hu Wt TCRβ gp100
Hu Wt TCRαβ pp65
Hu Wt TCRα gp100 + Hu Wt TCRβ pp65
MFIx 7.5
MFIx 5.7
MFIx 161
MFIx 178
MFIx 138
MFIx 163
Hu Pan TCR – PE
no Ab – APC
C
MFIx 7.1
MFIx 8.2
MFIx 226
MFIx 190
MFIx 98.9
MFIx 181
vβ 14 – PE
vβ 13.1 – PE
D
Hu Wt TCRα
Hu Wt TCRαβ gp100
(wild type 4-domain dcTCR)
Hu Chim TCRα
Hu Chim TCRαβ
Mu Cα
Hu Chim scTCR gp100 + Mu Cα
(3-domain scTCR + 1-domain Cα)
Hu Chim scTCR silCDR3α + Mu Cα
CDR3α S109Q
Hu Chim scTCR Vα-Li(Vβ) silCDR3α + Mu Cα
G49αC – GLi17C
T84C
S79C
Mu Wt TCRα
Mu Wt scTCR p53 + Mu Cα
(3-domain scTCR + 1-domain Cα)
Mu Wt scTCR silCDR3α + Mu Cα
CDR3α D109A
Mu Wt scTCR Vα-Li(Vβ) silCDR3α + Mu Cα
G51αC – GLi16C

**Supplementary Figure 1: Human Jurkat-76 cell line as a suitable model for molecular TCR interaction studies. (A)** Freshly OKT3-activated human T-cells as positive control and human leukemic cell line Jurkat-76 were un/stained with anti-human Pan TCR- or anti-human CD3-antibody. **(B/C)** 5 x $10^6$ Jurkat-76 cells were electroporated with 5µg RNA coding for TCRs of the gp100- or CMV pp65-specificity in wild type or hybrid combinations. TCR expression was analyzed with a Pan TCR-antibody (B) or with a subfamiliy-specific antibody **(C)** to stain for (mis)paired TCRaβ (Pan) or TCRβ (Vβ), respectively. The mean fluorescent intensity (MFI) for every specimen is given. TCRs were only expressed on the cell surface as wild type or hybrid TCR dimers, but not as monomeric chains. **Nomenclature and domain topology of un/modified TCR constructs used throughout this manuscript. (D)** The top panel depicts the domain arrangements of the wild type human TCRα chain of the gp100-specificity used as a 'sensor' for mispairing with a 3-domain scTCR of the same antigen-specificity and thus, served as a 'surrogate' for any endogenous TCRα chain in mispairing analyses. Additionally, the human wild type or in C-domain murinized (chimerized) double chain TCRs of the gp100- and pp65-specificity, respectively (green or grey). The middle panel depicts the autonomously coexpressed mouse Cα-domain (grey) along with modified human 3-domain single chain TCRs gp100 murinized in TCR C-domains (green-rimmed). They were either functionally unresponsive by a silencing mutation S109Q situated on top of the CDR3α antigen-recognizing loop, and/or stabilized in V-domain pairing via an artificial disulfide bond bridging Vα G49C with the C-terminal tail of the Gly/Ser-rich linker at position G17C. The bottom panel illustrates the wild type murine TCRα chain also used as a 'sensor' of mispairing and the modified murine single chain TCRs p53 (grey-rimmed) either functionally unresponsive by a silencing mutation D109A on top of CDR3α, and/or stabilized in V-domain pairing via an artificial disulfide bond bridging Vα G51C with the C-terminal tail of the same linker at position G16C. Beside the wild type disulfide bond in TCR C-domains a routinely used artificial disulfide bond between Cα T84C / Cβ S79C accomplished stronger C-domain interaction. Enumeration was according to the IMGT database as cited in the main text.

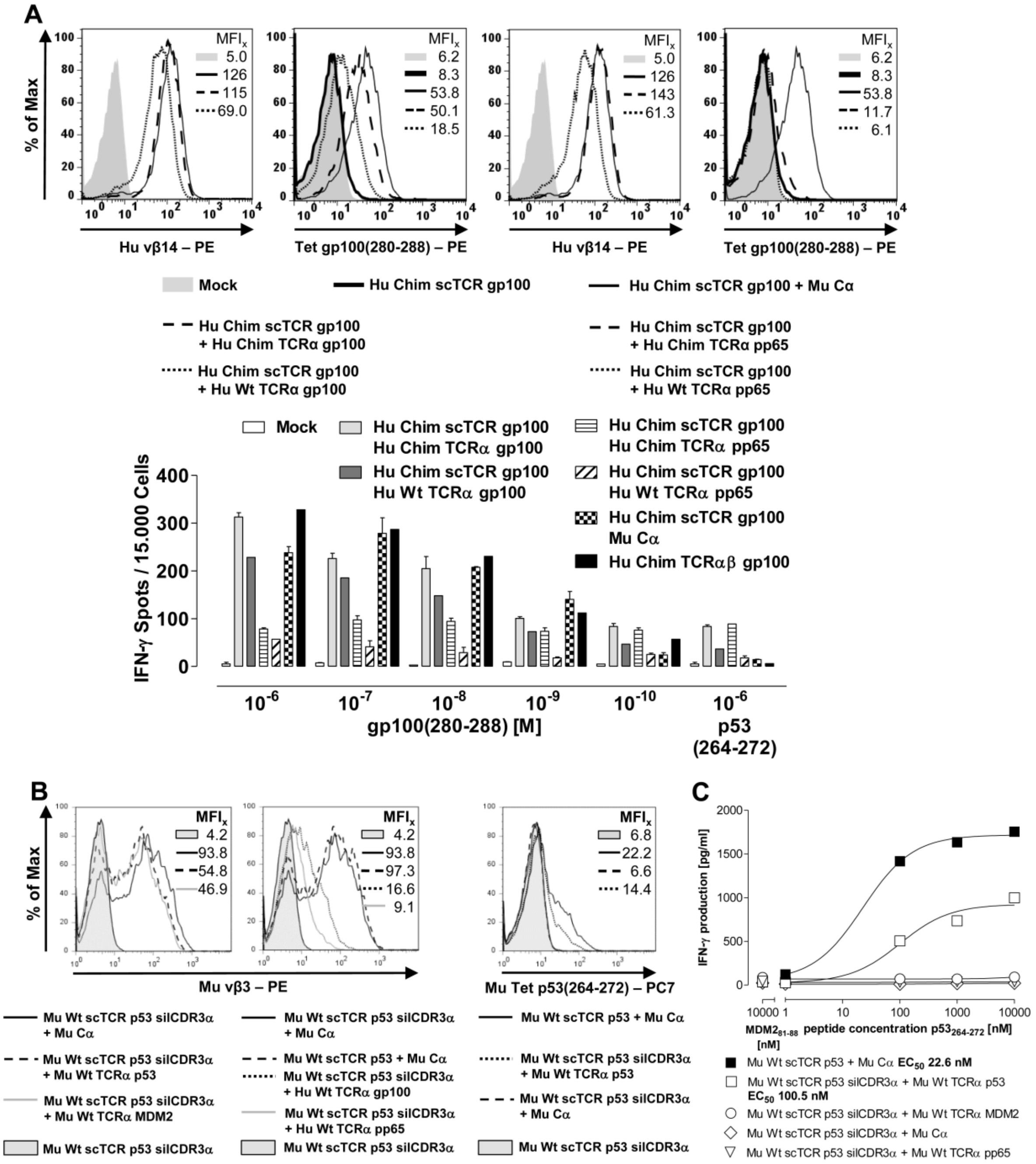


**Supplementary Figure 2: A 3-domain scTCR molecularly interacts with TCRα in human Jurkat-76 devoid of endogenous TCRs. (A) Murinization of human TCRα C-domains leads to higher mispairing with a chimerized human scTCR.** 5 x $10^6$ Jurkat-76 cells were electroporated with 5µg RNA coding for indicated TCR constructs. After 12 hours, TCR expression was analyzed cytofluorometrically by means of vβ14-staining or antigen recognition (top) as mentioned in Fig. 1. Additionally, the same responder cells were analyzed in IFN-γ spot production (below) in response to gp100(280-288) peptide-pulsed A2.1$^+$ T2 targets at the indicated peptide concentrations at an effector to target cell ratio of 0.3:1. As a control, T2 cells loaded with an A2.1-binding peptide of p53(264-272) were used. Data are shown as mean + SD of duplicates. A TCRα of the same antigen-specificity operates as a

‘sensor’ of mispairing and hence, as a ‘surrogate’ for any (endogenous) TCRα. Murinization in its C-domain facilitates TCRα-mispairing (readout by vβ14 and multimer) and, if coexpressed with an unrelated TCRα pp65, also TCR Cα-mispairing (readout by multimer). **Mispairing of a murine scTCR p53 with full length mouse or human TCRα-chains takes place in Jurkat-76.** (**B**) A 3-domain Wt or functionally unresponsive (silCDR3α) scTCR p53 construct was coexpressed with Mu Cα or diverse antigen (p53, MDM2, gp100, pp65)- and species (mouse, human)-un/related TCRα-chains as indicated. Each chain, encoded on a separate plasmid, was retrovirally introduced into J-76 and normalized in gene expression by drug selection, and expanded for at least a week. Expression of the TCR was analyzed by Vβ3- and antigen recognition by tetramer p53(264-272)-staining in flow cytometry. Mispairing of mouse scTCR p53 (Vβ3) with human TCRα (gp100, pp65) is much lower than mispairing with murine TCRα (p53, MDM2). Also, mispairing of a mouse scTCR p53 with human TCRα is much lower than mispairing of a human (chimerized) scTCR gp100 with human ones (Suppl. Fig. 2A). **(C)** The same panel of TCR p53 constructs introduced into J-76 was assessed for IFNγ-secretion after coculture with p53(264-272)-peptide-pulsed T2 cells dose-dependently at an E(Vβ3$^{+}$):T-ratio of 5:1. The half-maximal effective concentration ($EC_{50}$) is calculated from non-linear sigmoidal regression analysis and reflects the affinity of a given TCR for its cognate antigen. Functional data are shown as mean of duplicates.

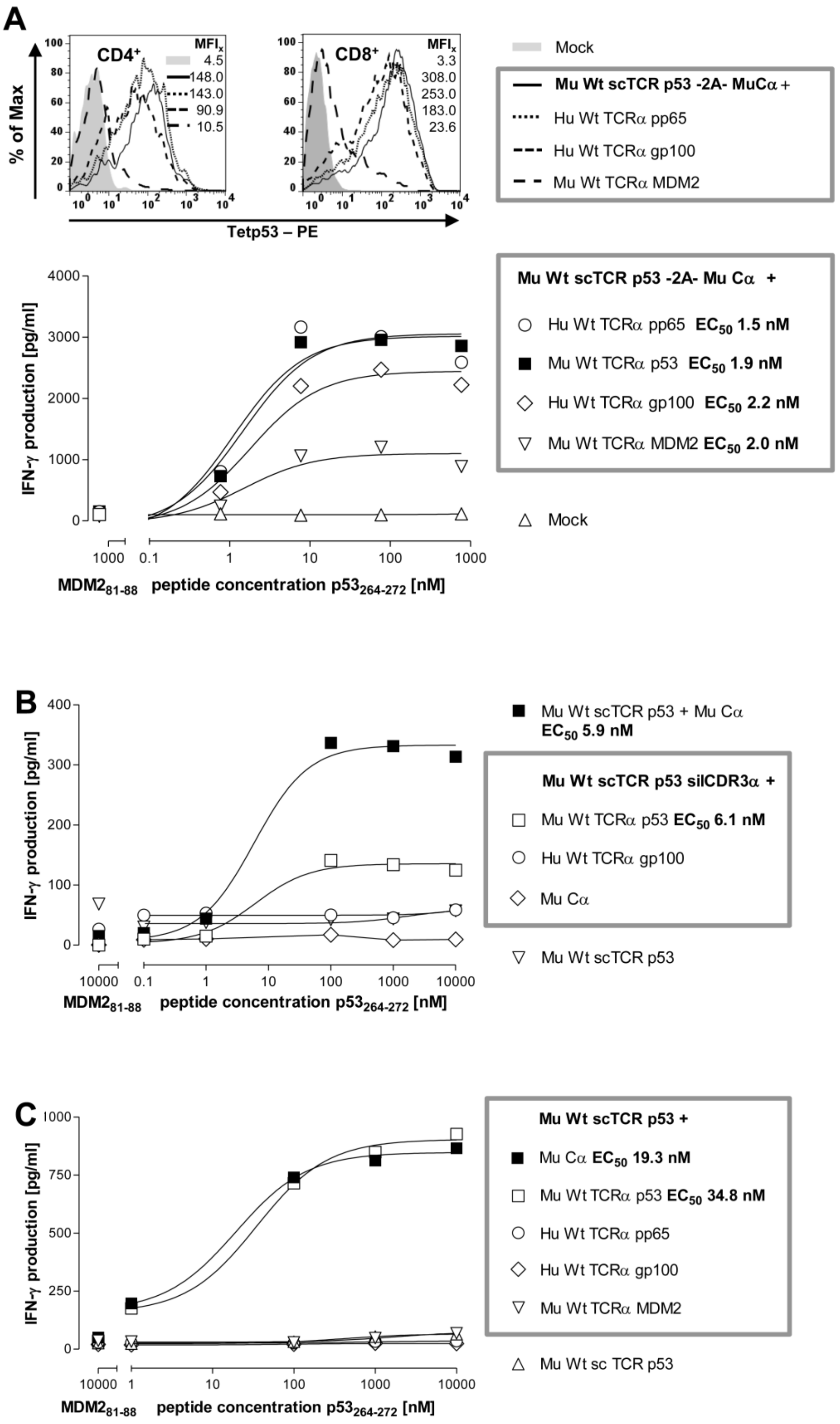

A
CD4+
CD8+
MFIx
4.5
148.0
143.0
90.9
10.5
3.3
308.0
253.0
183.0
23.6
% of Max
Tetp53 – PE
Mock
Mu Wt scTCR p53 -2A- MuCα +
Hu Wt TCRα pp65
Hu Wt TCRα gp100
Mu Wt TCRα MDM2
IFN-γ production [pg/ml]
Mu Wt scTCR p53 -2A- Mu Cα +
Hu Wt TCRα pp65 EC50 1.5 nM
Mu Wt TCRα p53 EC50 1.9 nM
Hu Wt TCRα gp100 EC50 2.2 nM
Mu Wt TCRα MDM2 EC50 2.0 nM
Mock
MDM2 81-88
peptide concentration p53 264-272 [nM]
B
Mu Wt scTCR p53 + Mu Cα
EC50 5.9 nM
Mu Wt scTCR p53 silCDR3α +
Mu Wt TCRα p53 EC50 6.1 nM
Hu Wt TCRα gp100
Mu Cα
Mu Wt scTCR p53
C
Mu Wt scTCR p53 +
Mu Cα EC50 19.3 nM
Mu Wt TCRα p53 EC50 34.8 nM
Hu Wt TCRα pp65
Hu Wt TCRα gp100
Mu Wt TCRα MDM2
Mu Wt sc TCR p53

**Supplementary Figure 3: Mispairing of a murine scTCR p53 with human TCRα takes place to less amounts in human T-cells. (A)** Bulk human T-cells were retrovirally transduced on separate plasmids with Wt scTCR p53-F2A-Cα-constructs, and TCRα-chains of the MDM2(81-88)-, gp100(81-88)-, or pp65(495-503)-specificity, and assayed for competition effects in flow cytometry analysis in $CD4^+$ (**left**) and $CD8^+$ (**right**) T-cell populations and in an IFNγ-ELISA at an (E)($CD8^+V\beta3^+$) : (T)-ratio of 0.1:1 (**below**). The half-maximal effective concentration ($EC_{50}$) is calculated from non-linear sigmoidal regression analysis and reflects the affinity of a given TCR for its cognate antigen. Data are shown as mean of duplicates. Competitive effects of TCRα for binding to scTCR follows the same order as observed in Jurkat-76 (Fig. 2B). **(B)** Bulk human T-cells were retrovirally transduced on separate plasmids with murine Cα, or Wt TCRα p53, or Wt TCRα gp100, and Wt or functionally unresponsive scTCR p53 (i.e. silCDR3α D109A), normalized in TCR expression via drug-selection, and expanded by a 2-weekly CD3/CD28-beads stimulation. They were analyzed for IFNγ-secretion in ELISA after coculture with peptide-pulsed T2 cells dose-dependently at an effector (E)($CD8^+V\beta3^+$) : target (T)-ratio of 0.3:1. TCRα-mispairing of a functionally unresponsive scTCR p53 with TCRα of the same antigen specificity was observed. **(C)** Bulk human T-cells were retrovirally transduced on separate plasmids with Wt scTCR p53 and TCRα-chains of the p53(264-272)-, MDM2(81-88)-, gp100(81-88)-, or pp65(495-503)-specificity and assayed as described in (A) at an (E)($CD8^+V\beta3^+$) : (T)-ratio of 0.1:1. None of the coexpressed antigen-unrelated TCRα chains, even the strongly binding murine TCRα MDM2, were able to reconstitute antigen recognition of a mouse scTCR p53 via TCR Cα-mispairing.

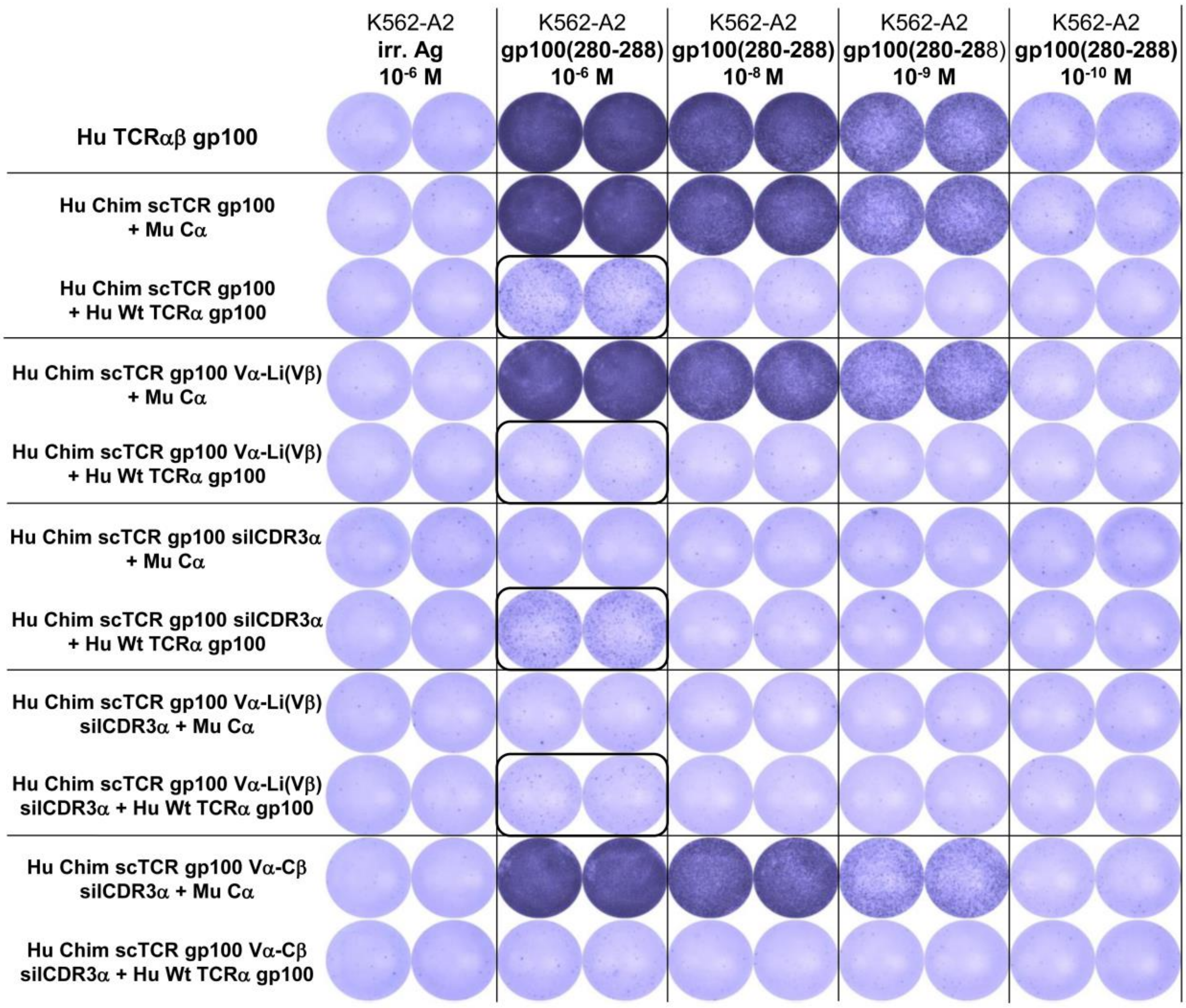


**Supplementary Figure 4: Prevention of residual mispairing in human T-cells by a novel artificial disulfide bond designed between Vα and the C-terminal tail of the linker close to Vβ for scTCR gp100.** 4-10 μg of RNA encoding Mu Cα, or TCRα gp100, or different scTCR gp100-constructs as described in Figures 4A, 6A/C were electroporated into MACS-purified human $CD8^+$ T-cells. Unmodified Hu Chim scTCR gp100 or functionally unresponsive Hu Chim scTCR gp100 silCDR3α S109Q were compared with their corresponding scTCR derivatives stabilized in the scTCR-fragment via the novel cystine bridge Vα-Li(Vβ). Normalized expression was assessed from coelectroporation of Mu Cα. TCRα-mispairing was assessed from coexpression with TCRα gp100. 20 h after coculture with K562-A2 dose-dependently pulsed with the relevant gp100(280-288) or an irrelevant peptide at $10^{-6}$M at an E:T-ratio of 6:1, responder T-cells were submitted to an IFNγ-Elispot-assay. The incorporation of the scTCR-fragment stabilizing disulfide bond into Hu Chim scTCR gp100 yielded equal IFNγ-spot production down to $10^{-9}$M peptide pulse compared

with unmodified Hu Chim scTCR gp100 + Mu Cα and importantly, eliminated residual IFNγ-spot production at $10^{-6}$M peptide pulse (black box) resulting from TCRα-mispairing.

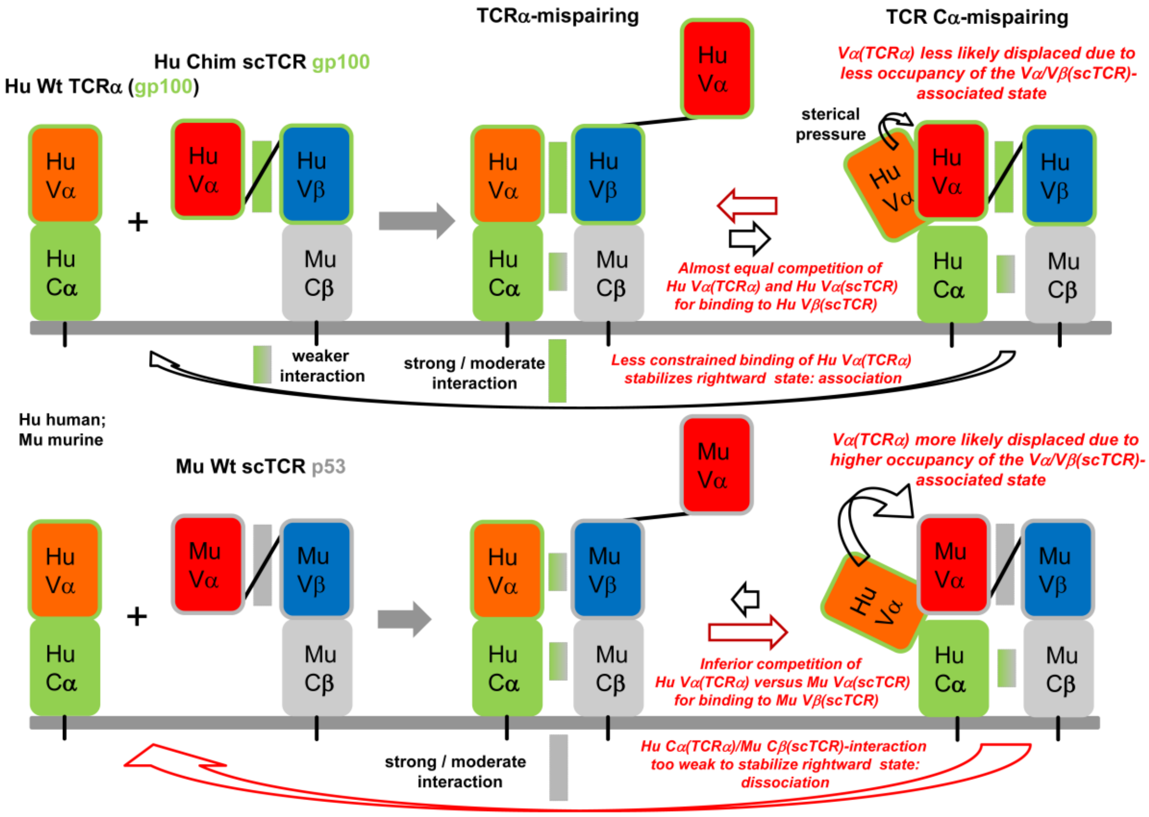


**Supplemental figure 5: TCRα- versus TCR Cα-mispairing and dissociation for human versus murine scTCRs.** The Vα-domain of any endogenous TCRα senses the presence of a human or mouse Vβ-domain in a given 3-domain single chain TCR. Competition between both Vα-domains (in TCRα vs scTCR) for binding to Vβ (in scTCR) results in either TCR Cα- versus TCRα-mispairing, a mixture thereof, or dissociation depending on relative interaction forces between Vα(TCRα)/Vβ(scTCR) and Vα(scTCR)/Vβ(scTCR), and Cα(TCRα)/Cβ(scTCR). (**Top**) In human T-cells a proportion of the vast excess of polyclonal endogenous TCRα appear to reconstitute antigen recognition of human Chim scTCR gp100 by TCR Cα-mispairing (Fig. 4A) in line with results in J-76 (Fig. 1A/B). Hypothetically, the intra-species Hu Vα(TCRα)-Hu Vβ(scTCR)-interaction successfully competes with a homologous intra-species Hu Vα(scTCR)-Hu Vβ(scTCR)-interaction. This leads to an almost equal occupancy of both states and provides sufficient ‘space’ for Hu Vα(TCRα) to be positioned close to Vβ(scTCR) in a sterically less constrained manner. Transient binding between Hu Vα(TCRα) and Hu Vβ(scTCR) contributes to the weaker inter-species binding between Hu Cα(TCRα) and Mu Cβ(scTCR) and thus, populates the state of TCR Cα-mispairing in equilibrium. (**Bottom**) Conversely, expression of a mouse scTCR p53 was largely stabilized by mouse TCRα (Fig. 4B/C)-, but not by human TCRα- or TCR Cα-

mispairing. In this case the weaker inter-species Hu Vα(TCRα)-Mu Vβ(scTCR)-interaction causes a shift towards the stronger intra-species Mu Vα(scTCR)-Mu Vβ(scTCR)-interaction which in turn does not sufficiently provide ‘space’ for Hu Vα(TCRα). The missing contribution of Hu Vα(TCRα) to chain pairing with a mouse scTCR leads to the dissociation of the weakly interacting inter-species Hu Cα(TCRα)/Mu Cβ(scTCR)-domains.

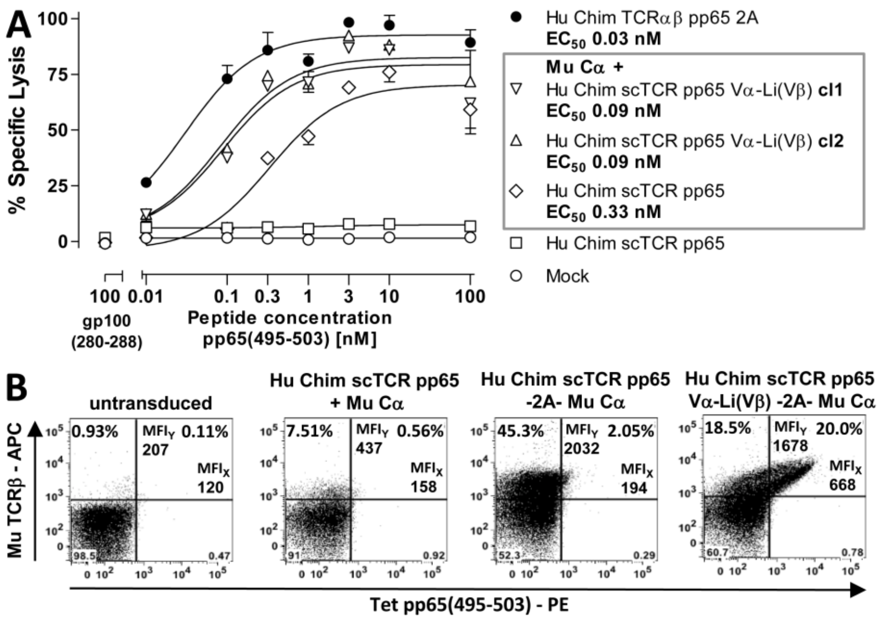


**Suppl. Figure 6: The novel Vα-Li(Vβ) disulfide bond improved functional and structural avidity of an unstable scTCR pp65. (A)** Cystine Vα-Li(Vβ)-stabilized Chim scTCR pp65 were tested for 2 independent plasmid clones cl1 and cl2 in a 51-chromium-based cytotoxicity assay in comparison with an unmodified Chim scTCR pp65 w/wo Mu Cα or a 2A-linked dcTCR pp65 chimerized with mouse C-domains, respectively. Retrovirally transduced T-cells were enriched and normalized for TCR expression by drug-selection and subsequently, were cocultured with dose-dependently pp65(495-503)- and 51-chromium-pulsed T2 cells at an $E(CD8^+)$:T-ratio of 10:1. Cytotoxicity was measured as the relative release of the radionuclide 51-chromium following T-cell dependent target cell killing. This is one out of 2 representative assays. Data are shown as mean + SD of duplicates. **Cystine-modification of an unstable scTCR pp65 triggered multimer-binding. (B)** Chim scTCR pp65 was either coexpressed with Mu Cα on separate retroviral plasmids (pMP71) in bulk human T-cells or Chim scTCR pp65 without and with the stabilizing disulfide bond Vα-Li(Vβ) were linked to Mu Cα on a single retroviral construct via the self-processing peptide 2A. Transduced T-cells were restimulated with irradiated pp65(495-503) peptide-loaded T2 cells and feeder PBMCs in 10-12 days intervals two times. Multimer-staining was assessed with a pp65(496-503)-specific tetramer. At that time, the frequency of $CD8^+$ T-cells was almost the same in the range of approximately 90% for all modified T-cells depicted here (not shown).
